# Diterpenoids in medicinal plants: structure, distribution, biological activities, biosynthesis, and bioengineering prospects

**DOI:** 10.3389/fpls.2026.1833614

**Published:** 2026-05-19

**Authors:** Xinqi Zou, Yunqing Cheng, Xuefei Chen

**Affiliations:** Provincial Key Laboratory of Plant Resource Science and Green Production, Jilin Normal University, Siping, China

**Keywords:** bioengineering, biosynthesis, distribution, diterpenoids, medicinal plants, pharmacological activities

## Abstract

Diterpenoids are important natural secondary metabolites characterized by a 20-carbon backbone and classified into acyclic, monocyclic, bicyclic, tricyclic, tetracyclic, and macrocyclic types based on their ring structures. Abundant in medicinal plants, these compounds function in defense against pathogens and herbivores and exhibit diverse pharmacological activities, including anticancer, anti-inflammatory, cardiovascular-protective, antidiabetic, and antimicrobial effects. The biosynthetic precursor (E,E,E)-geranylgeranyl diphosphate (GGPP) is primarily generated through the methylerythritol 4-phosphate (MEP) pathway in plants. The carbon skeleton of diterpenoids is then constructed by diterpene synthases (diTPSs) and undergoes diverse modifications catalyzed by cytochrome P450 monooxygenases (CYP450s), alcohol dehydrogenases (ADHs), and UDP-dependent glycosyltransferases (UGTs), while transcription factors orchestrate pathway gene expression. Advances in synthetic biology and metabolic engineering have enabled heterologous production of diterpenoids in genetically tractable hosts, with yields enhanced through promoter optimization, targeted mutagenesis, and fermentation optimization. This review systematically summarizes the structure, distribution, biological activities, and biosynthetic pathways of diterpenoids, and discusses future directions including structural modification for enhanced bioactivity, pathway elucidation via omics analysis, modular biosynthesis for overproduction, and machine learning applications in pharmacology and enzyme engineering. These efforts provide a foundation for discovering novel bioactive diterpenoids, elucidating complete biosynthetic pathways, and enabling sustainable production through biotechnological breeding and synthetic biology.

## 1 Introduction

Diterpenoids are important natural secondary metabolites and represent a subclass of terpenoids characterized by a 20-carbon (C20) backbone. Based on their ring structures, they can be classified into acyclic, monocyclic, bicyclic, tricyclic, tetracyclic, and macrocyclic types (Liu et al., 2023). Diterpenoids are abundant in medicinal plants, where they function in defense against pathogens and herbivores (Wang and Wang, 2025). In addition, these compounds display a wide range of pharmacological activities, such as anticancer, anti-inflammatory, cardiovascular-protective, antidiabetic, and antimicrobial effects (Wang et al., 2025b). Given that dietary intake of diterpenoids is closely associated with human health, their extensive development and application hold considerable significance.

The biosynthetic precursor of diterpenoids, (E,E,E)-geranylgeranyl diphosphate (GGPP), is primarily generated through the methylerythritol 4-phosphate (MEP) pathway in plants (Laule et al., 2003; Zhao et al., 2013; Bergman et al., 2019). GGPP is subsequently cyclized and rearranged by diterpene synthases/cyclases (diTPSs) to generate over 100 distinct diterpene carbon skeletons (Hu et al., 2021). Based on these skeletons, medicinal plants produce a wide array of diterpenoid compounds through modifications catalyzed by various enzymes, such as cytochrome P450 monooxygenases (CYP450s), alcohol dehydrogenases (ADHs), and UDP-dependent glycosyltransferases (UGTs) (Bathe and Tissier, 2019). Furthermore, transcription factors (TFs) play crucial regulatory roles in diterpenoid biosynthesis by orchestrating the expression of multiple key genes in plant secondary metabolism (Zhou et al., 2024).

With the advancement of synthetic biology and metabolic engineering, heterologous synthesis of plant natural products in genetically tractable hosts—such as *Escherichia coli*, *Saccharomyces cerevisiae*, and *Nicotiana benthamiana*—has emerged as a promising alternative for large-scale production (Park et al., 2020; Li et al., 2023). Production yields can be further enhanced through multifaceted engineering approaches, including the introduction of synthetic and key upstream genes, promoter optimization and targeted mutagenesis to improve enzyme activity, modulation of TFs and transporters, and the implementation of optimized fermentation conditions.

In this review, we systematically summarize and analyze the structure, distribution, biological activities, biosynthetic pathways, and bioengineering applications of diterpenoids. We further discuss key areas of progress and future directions, including: (i) enhancing biological activities through mechanistic insights and structural modification; (ii) pathway elucidation via omics analysis and large-scale screening; (iii) diterpenoid overproduction using modular biosynthesis and multifaceted engineering at the genetic, enzyme, cellular, and fermentation levels; and (iv) leveraging machine learning as an efficient platform for pharmacology, biosynthesis, and enzyme engineering. Collectively, these efforts provide a valuable reference for the large-scale synthesis, development, and application of diterpenoids, while offering new insights into the future biomanufacturing of natural products.

## 2 Structure and distribution of diterpenoids

### 2.1 Structure of diterpenoids

**Figure 1:**
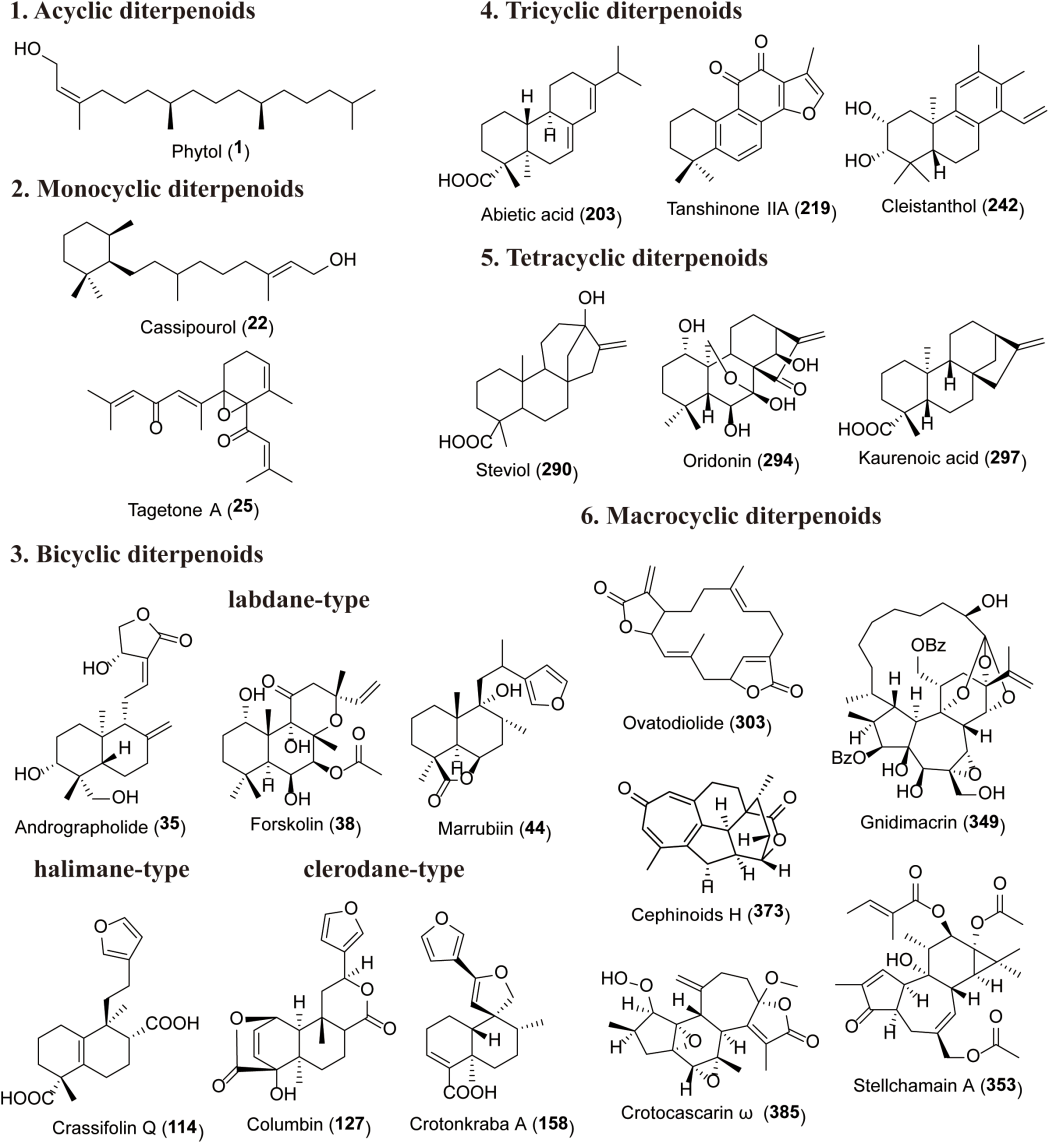
Structure of the representative diterpenoids of various rings.

Diterpenoids are composed of four C5 isoprene units linked in a head-to-tail manner, resulting in a fundamental skeleton of 20 carbon atoms. Based on their structural characteristics, diterpenoids can be mainly classified into acyclic, monocyclic, bicyclic, tricyclic, tetracyclic, and macrocyclic types (**Figure 1**; **Table 1**; **Supplementary Figure 1**). Certain types, such as cephalotane, daphnane, grayanane, tigliane, and crotofolane, contain a 7-membered ring and also feature three or four rings (Zhao et al., 2025b). However, because their biosynthesis is considered to originate from macrocyclic precursors (Luo et al., 2016), these types are discussed within the macrocyclic group in this review.

**Table 1:** Distribution and pharmacological activity of diterpenoids.

Compounds	Species	Bioactivities	References
1. acyclic diterpenoids			
Phytol (1)	algae, plants and cyanobacteria	Anticancer, antinociceptive, anti-inflammatory, antimicrobial: *E. coli*, *P. aeruginosa* (MIC = 62.5, 19 μg/mL)	(Islam et al., 2015, 2018)
Geranyllinalool (2)	*Tripterygium wilfordii, Gossypium hirsutum*	Anti-insect	(Su et al., 2017; Liu et al., 2018a)
Capsianosides I, IV, V (3-5)	*Capsicum* spp.	Anticancer, defense-related responses in plants	(Chilczuk et al., 2020)
Lipskynoids A-G (6-12)	*Carpesium lipskyi*	Anti-inflammatory: LPS-stimulated RAW 264.7 cells (IC_50_ = 9.9-18.47 μM)	(Zhong et al., 2022)
(13,14)	*Toona sinensis*	Antidiabetic: increased Nrf-2/HO-1 and decreased NF-κB, TNF-α and IL-6 in GMCs (30 μM)	(Chen et al., 2022)
(15-17)	*Aphanamixis polystachya*	Anti-inflammatory: LPS-stimulated RAW 264.7 cells (IC_50_ = 17.6, 9.8, 16.6 μM)	(Xue et al., 2020)
(18-20)	*Eupatorium lindleyanum*		(Wang et al., 2020)
(21)	*Vernicia montana*	Anti-inflammatory: LPS-stimulated BV-2 cells (IC_50_ = 3.18 μM)	(Wang et al., 2022)
2. monocyclic diterpenoids			
Cassipourol (22)	*Dacrycarpus imbricatus*	Anticancer: decreased OCI-AML cell number (60, 70 μg/mL)	(Tam et al., 2019)
Sauruchinenols A, B (23,24)	*Saururus chinensis*		(Gao et al., 2017)
Tagetones A, B (25,26)	*Tagetes minuta*	Anticancer: 25: MCF7, A549 cells (IC_50_ = 4.68, 4.24 μM); 26: HCT116 cells (IC_50_ = 6.30 μM)	(Ibrahim and Mohamed, 2017)
Solanerioside A (27)	*Solanum erianthum*		(Peng et al., 2017)
Trixagol (28), (29)	*Rosmarinus officinalis*	Antimicrobial: *B. subtilis, S. aureus, P. aeruginosa*, \ *Fusarium* spp. (MIC = 1-64 μg/mL)	(Xu et al., 2025)
(30)	*Clerodendrum trichotomum*		(Xu et al., 2015)
3. bicyclic diterpenoids			
Cuceolatins A-C (31-33)	*Cunninghamia lanceolata*	Antimicrobial: *B. subtilis, S. aureus* (IC_50_ < 25 μM)	(Yu et al., 2019)
Isocupressic acid (34)	*Juniperus polycarpos*	Anticancer: HepG2 cells (IC_50_ = 3.73 μg/mL)	(Naderian et al., 2025)
Andrographolide (35)	*Andrographis paniculata*	Anticancer, anti-inflammatory, antimicrobial, antidiabetic	(Tundis et al., 2023)
Manool, sclareol (36, 37)	*Salvia tingitana*	Antimicrobial: 36: streptococci (MIC = 4–32 mg/mL)	(Bisio et al., 2020)
Forskolin (38)	*Coleus forskohlii*	Antimicrobial: HAV, COX-B4, HSV-1, HSV-2 (IC_50_ = 62.9, 73.1, 99, 106 μg/mL)	(Amen et al., 2024)
(39-43)	*Rosmarinus officinalis*	Antimicrobial: 41: *B. subtilis*, *S. aureus* and *P. aeruginosa* (MIC = 0.5, 4, 128 μg/mL); 42: *B. subtilis* and *P. aeruginosa* (MIC = 0.125, 1 μg/mL)	(Xu et al., 2025)
Marrubiin (44)	*Lagopsis supina* *Marrubium vulgare*	Anti-inflammatory: reduced CTSC and NSPs activities in blood and bone marrow in mice without toxicity (IC_50_ = 30 mg/kg)	(Zhou et al., 2025a)
Scoparicol E (45)	*Scoparia dulcis*	Antidiabetic: reduced MLD-STZ-induced hyperglycemia in mice	(Dong et al., 2024)
Salvic acid (46)	*Eupatorium salvia*	Antimicrobial: *S. aureus* and *B. cereus* (MIC = 50 mg/mL)	(Echeverria et al., 2017)
Forsypensins A-E (47-51)	*Forsythia suspensa*	Anti-inflammatory, antimicrobial: inhibited β-glucuronidase release by 43.6%-49.2% in rat PMNL (10 μM); H1N1 virus and RSV (IC_50_ = 21.8-27.4 μM, EC_50_ = 10.5-15.4 μM)	(Xiang et al., 2020)
(52-61)	*Hedychium ellipticum*	Anticancer, antimicrobial: 52, 58: *M. tuberculosis* (MIC = 12.5, 6.25 mg/mL); 55, 57, 58: NCI-H187 cells (IC_50_ = 0.12, 0.90, 0.72 μg/mL)	(Songsri and Nuntawong, 2016)
cis-Abienol (62)	*Nicotiana* spp.	Antimicrobial: induced tomato resistance against bacterial wilt	(Zhang et al., 2025b)
Sublyratins A-O (63-77)	*Croton sublyratus*	Anticancer: 71: HL-60 cell (IC_50_ = 2.8 μM)	(Qi et al., 2021)
Crotonoids A–H (78-85),(86)	*Croton Sublyratus*	Anti-inflammatory: 86: LPS-stimulated RAW 264.7 cells (IC_50_ = 10.02 μM)	(Qi et al., 2024)
(87-91)	*Croton laevigatus*	Antidiabetic: 88: inhibited PTP1B (IC_50_ = 4.11 μg/mL)	(Liu et al., 2018b)
Lauenones A, B (92, 93)	*Croton laui*	Anti-adipogenic: inhibited adipogenesis in 3T3-L1 cells (EC_50_ = 13.07-42.57 μM)	(Zhang et al., 2025c)
Launines A-I (94-102)	*Croton laui*	Anti-inflammatory: 94, 97, 100, 101: LPS-stimulated RAW 264.7 cells (IC_50_ = 42.73-93.04 μM)	(Yang et al., 2016)
(103-113)	*Vitex trifolia*	Anticancer: 110: HCT 116 cells (IC_50_ = 20.3 µM)	(Luo et al., 2017)
Crassifolins Q-W (114-120)	*Croton crassifolius*	Anti-inflammatory, anti-angiogenesis: inhibited IL-6 and TNF-α in LPS-stimulated RAW 264.7; 118: inhibited adipogenesis in HUVECs (6.25-50 μM)	(Li et al., 2021)
Crapenoids A-C (121-123)	*Croton crassifolius*	Anti-inflammatory: 121: LPS-stimulated RAW 264.7 cells (IC_50_ = 25.8 μM)	(Ye et al., 2021)
Crassifolius A-C (124-126)	*Croton crassifolius*	Anticancer: 124: Hep3B, HepG2 cells (IC_50_ = 17.91, 42.04 μM)	(Tian et al., 2017)
Columbin, tinocapillins A–C, tinocallones A-C (127-133)	*Tinospora capillipes*	Anticancer: 128, 129, 133: A549, Hep G2, Hela, OS-RC-2 cell (IC_50_ = 9.6-19.1 μM)	(Zhan et al., 2009; Wang et al., 2016a)
Tinosinoids A-J (134-143)	*Tinospora sinensis*	Antidiabetic, anticancer: 140, 141: inhibitied α-glucosidase (IC_50_ = 46.0, 29.7 μM); 134, 141: HCT-116, HeLa (IC_50_ = 19.1-48.2 μM)	(Zhang et al., 2022)
(144-151)	*Casearia graveolens*	Anticancer: 151: inhibited tumor proliferation and metastasis in *zebrafish xenograft*	(Wang et al., 2024a)
Crocleropenes A, B (152, 153)	*Croton caudatus*	Anticancer: MCF-7 cells (IC_50_ = 35.8-40.2 μM)	(Zou et al., 2020)
Croforins A-D (154-157)	*Croton foribundus*	Anti-inflammatory: 154, 155, 157: LPS-stimulated RAW 264.7 cells (IC_50_ = 25.47-40.26 μM)	(Queiroz et al., 2020)
Crotonkrabas A-C (158-160)	*Croton krabas*	Antimicrobial: 158, 159: *B. cereus*, *B. subtilis* (MIC = 64, 128 μg/mL)	(Rajachan et al., 2021)
Crotonines A-H (161-168)	*Croton yunnanensis*	Antidiabetic: 161, 166: enhanced glucose uptake in insulin-resistant 3T3-L1 cells (20 μM)	(Jiang et al., 2023b)
(169-181)	*Salvia guevara*	Anticancer, anti-inflammatory: 170, 175: K562 cells (IC_50_ = 33.1, 39.8 μM); 174, 175, 178: LPS-stimulated RAW 264.7 cells (IC_50_ = 26.4, 17.3, 13.7 μM)	(Torres-Médicis et al., 2025)
Calintegerinoids A-E (182-186)	*Callicarpa integerrima*	Anti-inflammatory: 184: inhibitied LDH release (IC_50_ = 1.27 μM), blocked NLRP3 inflammasome activation triggered by LPS and Nigericin	(Zeb et al., 2025)
Forsyditerpenes A-L (187-198)	*Forsythia suspensa*	Anti-inflammatory: 189, 190: LPS-stimulated RAW 264.7 cells (IC_50_ = 26.2, 12.8 μM)	(Li et al., 2025a)
Baccharisacetals A,B, epibaccharisacetals A, B (199-202)	*Baccharis trimera*	Hepatoprotective: againsted alcohol-induced hepatic injury in LX-2 cells and in *z. xenograft*	(Liang et al., 2026)
4. Tricyclic diterpenoids			
Abietic acid (203)	*Pinus palustris*, *P. abies, Pimenta racemose*	Antimicrobial, anti-inflammatory: *Streptococcus mitis* (MIC = 16 mg/mL); anti-inflammatory via COX-2 inhibition and PPARα/γ activation	(Ito et al., 2020; Ren et al., 2026)
Ferruginol, miltiodiol (204, 205)	*Perovskia abrotanoides*	Antiparasitic: 204: *Plasmodium falciparum* (IC_50_ = 2.9 µM); 205: *Trypanosoma brucei rhodesiense* (IC_50_ = 0.5 µM)	(Tabefam et al., 2018)
(206-217)	*Clerodendrum trichotomum*	Anti-inflammatory: 206, 209, 212: LPS-stimulated RAW 264.7 cells (IC_50_ = 6-10.6 μM)	(Hu et al., 2018)
Tanshinone I, tanshinone IIA, cryptotanshinone (218-220)	*Salvia miltiorrhiza*	Anti-inflammatory, anti-vascular disease	(Li et al., 2025b)
(221-223)	*Croton lachnocarpus*	Anticancer: 223: A-549, BGC-823, HepG2, HL-60, MCF-7, W480 (IC_50_ = 24.9-29.4 μM)	(Wang and Zhang, 2023)
(224-226)	*Croton cascarilloide*	Antimicrobial: *Corynebacterium*, *E. faecalis*, *Enterococcus* sp. (MIC < 50 μg/ml)	(Wang and Dong, 2023)
Mangiolide (227), (228-231)	*Suregada zanzibariensis*	Antimicrobial, antiparasitic: 227: *P. falciparum* (IC_50_ = 0.79, 0.87 µg/mL), *C. neoformans*, *S. aureus*, *E. faecium* (IC_50_ = 1.20, 3.90, 7.20 µg/mL)	(Matundura et al., 2023)
Euphorfinoids E-L (232-239)	*Euphorbia fischeriana*	Acetylcholinesterase inhibitory: 232: (IC_50_ = 6.23 μM)	(Wei et al., 2021)
Alpinoblonoids A,B (240,241)	*Alpinia oblongifolia*	Hepatoprotective: inhibited the expressions of fibronectin, collagen I, α-smooth muscle actin.	(Zhang et al., 2025a)
Cleistanthol, sauspatulifols A-L (242-254)	*Sauropus spatulifolius*	Antimicrobial: 242, 247: *S. aureus*, *S. epidermidis*, *M. luteus*, *B. subtilis*, *S. flexneri*, *S. pneumoniae* (MIC = 12-32 µg/mL)	(Wu et al., 2022)
Icacinlactones A-G (255-261)	*Icacina trichantha*	Anticancer: 260: MDA-MB-435, MDA-MB-231, OVCAR3 cells (IC_50_ = 6.16, 8.94, 10.5 μM)	(Zhao et al., 2015)
(262-264)	*Croton niveusJacq*	Anticancer: 262, 263: HCT-15, PC-3 cells (IC_50_ = 34.76-55.68 μM)	(Reyes-Hernández et al., 2023)
Phyllanfranins A-F (265-270)	*Phyllanthus franchetianus*	Anti-inflammatory: 268: LPS-stimulated RAW 264.7 cells (IC_50_ = 19.03 μM)	(Qin et al., 2025)
Gopherenediol, lathyrisone A, spirolathyrisins B-D (271-275)	*Euphorbia lathyris*	Antimicrobial: *F. oxysporum*, *A. alternata*, *B. dothidea*, *D. sojae* (EC_50_ = 7.73-14.81 µg/mL)	(Wang et al., 2024c)
(276-281)	*Caesalpinia minax*	Anticancer: 281: MCF-7, HEY, A549 cells (IC_50_ = 8.0, 10.74, 25.34 µM)	(Xu et al., 2022b)
Caeminaxins A, B (282, 283)	*Caesalpinia minax*	Anti-inflammatory: LPS-stimulated BV-2 cells (IC_50_ = 10.86, 12.76 µM)	(Lu et al., 2023)
Taepeenin D (284)	*Caesalpinia mimosoides*	Anti-renal fibrosis: regulated the expression of E-cadherin, α-SMA, collagen I, fibronectin	(Wang et al., 2025a)
Caesalminines C - G (285-289)	*Caesalpinia bonduc*	Anticancer: A431, A549, U87MG cells	(Yan et al., 2024)
5 Tetracyclic diterpenoids			
Steviol (290)	*Stevia rebaudiana*	Antidiabetic	(Pelczynska et al., 2025)
Isosteviol (291)	*Stevia rebaudiana*	Antimicrobial, anticancer, antidiabetic: HCT116 cells (IC_50_ = 24.8 μM)	(Chen et al., 2025c; Zhou et al., 2025b)
(292, 293)	*Baccharis retusa*	Antiparasitic: *T. cruzi* (IC_50_ = 3.8, 44.2μM)	(Ueno et al., 2018)
Oridonin (294)	*Rabdosia rubescens*	Anticancer, anti-inflammatory: inhibited tumor growth in A549 cell-derived xenograft nude mice	(Han et al., 2025; Shi et al., 2026)
Crotonmekongenin A (295)	*Croton mekongensis*	Anticancer: FaDu, HT-29, SH-SY5Y (ED_50_ = 0.48, 0.63, 0.45 µg/ml).	(Udomthawee et al., 2021)
Trigoheterone A (296)	*Trigonostemon heterophyllus*	Anticancer: HL-60, SMMC-7721, A549, MCF-7, SW480 (IC_50_ = 2.05-0.86 μM)	(Leonelli et al., 2021)
Kaurenoic acid (297)	*Copaifera reticulata*	Antimicrobial: *E. faecium* and *S. aureus* (IC_50_ = 2.3, 3.4 μg/mL)	(Çiçek et al., 2020)
Rhodomollein LII, LIII (298, 299)	*Rhododendron molle*	Antinociceptive: 299: inhibited 89.0% by an acetic acid-induced writhing test (20 mg/kg)	(Li et al., 2020)
6. Macrocyclic diterpenoids			
Cembratriene-ol (300)	*Nicotiana* spp.	Antimicrobial, anti-insect: destroyed the endometrial structures of fungi, inhibited the growth of *Valsa mali* (80 μg/mL)	(Xu et al., 2024)
EBC-304, EBC-320 (301, 302)	*Croton insularis*	Anticancer: HeLa, HT-29, MACF-7, MM96L, K562, NFF (IC_50_ = 3-7 μM)	(Maslovskaya et al., 2017)
Ovatodiolide (303)	*Anisomeles indica*	Anticancer: effected NF-*κ*B/MMP-9, JAK2/STAT3, PI3K/AKT/mTOR, Wnt/*β*-catenin	(Zhou et al., 2026)
Euphjatrophanes H–L (304-308) (309-316)	*Euphorbia peplus*	Increase autophagic flux: 304, 315: displayed BBB permeability (log*Pe* = −4.853, −5.017)	(Chen et al., 2025a)
Euphzycopins A-D (317-320)	*Euphorbia Helioscopia*	Anti-inflammatory: 320: inhibited NLRP3 inflammasome (IC_50_ = 7.75 μM)	(Qiu et al., 2024b)
(321-330)	*Croton damayeshu*	Anti-insect: 328-330: *Plutella xylostella* (LC_50_ = 0.19, 0.16, 0.26 μM)	(Jiang et al., 2022)
Crotignoids A-K (331-241)	*Croton tiglium*	Anticancer: 331: HL-60 (IC_50_ = 1.61 μM)	(Zhang et al., 2015)
Fischerianins A, B (342, 343)	*Euphorbia fischeriana*	Anticancer: HL-60, K562 cells (IC_50_ = 12.82-17.82 μM)	(Xie et al., 2021)
Malleatins A, B (344, 345)	*Euphorbia malleata*	Anticancer: 344: A2780 wild, A2780 R-CIS cells (IC_50_ = 50-65 μM)	(Zolfaghari et al., 2024)
Kopetdaghinanes A, B (346, 347)	*Euphorbia kopetdaghi*	Anticancer: MCF-7, OCVAR-3 cells (IC_50_ = 38.10, 51.23 μM)	(Riahi et al., 2020)
Jatrophodione A (348)	*Euphorbia Macrorrhiza*	Anticancer: KB, KBv200 cells (IC_50_ = 22.46, 47.87 μM)	(Gao et al., 2016)
Gnidimacrin, daphneodorins A-C (349-352)	*Daphne odora*	Antimicrobial: 349-351: inhibited HIV-1 replication (EC_50_ = 0.061, 0.16, 0.25 nM)	(Otsuki et al., 2020)
Stellchamain A (353)	*Stellera chamaejasme*	Antipsoriasis: reduced IMQ-induced epidermal thickness, hyperkeratosis, and perivascular inflammatory cell infiltration *in vivo*	(Nie et al., 2025)
Pierisformosoids A-L (354-365)	*Pieris formosa*	Antinociceptive, anti-insect: 354, 355, 357, 358, 360, 361: analgesia in an acetic acid-induced writhing test (5.0 mg/kg); 354, 357, 362: antifeedant in *Plutella xylostella* (0.5 mg/mL)	(Niu et al., 2018)
Cephinoids A-S (366-384)	*Cephalotaxus fortune, C. lanceolata*	Anticancer, anti-inflammatory: A549, HeLa and SGC-7901 cells (IC_50_ = 0.1-20 μM); 373: inhibited 49.0% on A549 cells in zebrafish (60.0 ng/mL)	(Ni et al., 2018; Ge et al., 2019)
Crotocascarin ω (385)	*Croton dichogamus*	Antimicrobial, anticancer: inhibited HIV-1 replication (IC_50_ = 5.3 nM); MT-4 cells (IC_50_ = 84 µM)	(Terefe et al., 2023)
Crotokilwaepoxides A-F (386-391)	*Croton kilwae*	Antimicrobial, antiparasitic: 387, 390: HRV-2 (IC_50_ = 44.6, 44.6 μM); 386-388: inhibited plasmodial 80–100% (50 μM)	(Mahambo et al., 2023)
Strophiofimbrins A, B (392, 393)	*Strophioblachia fimbricalyx*	Anticancer: Hela, A549, HepG2 (IC_50_ = 6.74-20.34 μM)	(Jiang et al., 2023a)
Dimorpoloids A-D (394-397)	*Dimorphocalyx poilanei*	Anti-adipogenic: 394, 396, 397: inhibited adipogenesis in 3T3-L1 cells (EC_50_ = 5.85-16.69 μM)	(Chen et al., 2025b)
Jatromultones A-I (398-406)	*Jatropha multifida*	Anticancer: 401: A549, HeLa, HepG2, MDA-MB-231, HepG2/DOX (IC_50_ = 2.69 to 5.8 μM)	(Zhang et al., 2018; de Souza et al., 2024)

Acyclic diterpenoids (1-21), also known as linear diterpenoids, typically possess an unsaturated straight-chain structure. They often serve as precursors for the biosynthesis of other compounds and are generally unstable, with relatively few types found in nature. A representative example is phytol (1), an acyclic unsaturated diterpenoid that serves as a component of the chlorophyll molecule (Islam et al., 2015, 2018). It is produced by nearly all photosynthetic organisms, including algae, plants, and bacteria (cyanobacteria). Monocyclic diterpenoids (22-30) contain a five- or six-membered ring, which is typically located at the terminal end of the molecule but may occasionally appear in the middle (25-26). This type of compound is primarily found in animal livers, the fungus *Schizophyllum commune*, and the sponge *Sarcotragus* sp (Xu et al., 2023; Wang et al., 2024b), and have also been documented in certain species of the Podocarpaceae, Saururaceae, Solanaceae, Lamiaceae, and Asteraceae.

Bicyclic diterpenoids are characterized by a carbon skeleton containing two ring structures, and over 10,000 distinct bicyclic diterpenoids have been identified to date (Wang et al., 2025b). This group in plants primarily comprises three subclasses: labdanes, halimanes, and clerodanes. Labdane-type diterpenoids (31-37, 41-108) feature a bicyclic decalin ring system (C-1 to C-10) with a six-carbon side chain attached at C-9 (C-11 to C-16). The remaining four methyl groups (C-17 to C-20) are attached to the decalin skeleton at C-4 (geminal dimethyl), C-8, and C-10, respectively. Among this class, the most extensively studied compound is andrographolide (35), which has been demonstrated to possess anticancer, anti-inflammatory, antibacterial, neuroprotective, hepatoprotective, hypoglycemic, and immunomodulatory properties in various *in vitro* and *in vivo* disease models (Abu-Ghefreh et al., 2009; Tundis et al., 2023). Halimane-type bicyclic diterpenoids (39, 109-111, 114-126) represent a relatively small class of natural products. From a biosynthetic perspective, halimanes are considered intermediate types between labdanes and clerodanes (Zhao et al., 2025b). Clerodane-type diterpenoids (127-202) share a basic skeleton similar to that of labdanes, featuring a bicyclic decalin ring system (C-1 to C-10) with a six-carbon side chain attached at C-9 (C-11 to C-16). Typically, the remaining four methyl groups (C-17 to C-20) are attached to the decalin skeleton at C-4, C-5, C-8, and C-10.

Tricyclic diterpenoids (203-289) feature a carbon skeleton containing three ring structures, typically with 6/6/6 or 6/5/6 frameworks. The predominant tricyclic diterpenoids in plants are abietanes, along with cleistanthanes, rosanes, and cassanes. Abietanes (203-239) possess a basic skeleton of a normal series tricyclic perhydrophenanthrene framework, in which the methyl group (C-20) attached to C-10 is always β-oriented, two methyl groups (C-18 and C-19) are attached to C-4, and an isopropyl group is attached to C-13 (Wang and Dong, 2023). Notable examples include abietic acid (203), which exhibits strong antibacterial activity (Kameyama et al., 2020), and tanshinone IIA (218), which is used in the treatment of cardiovascular disease (Li et al., 2025b). Cassanes (276-289) are a group of tricyclic diterpenoids isolated from plants of the genus *Caesalpinia* (Fabaceae), characterized by the presence of a furan ring or an α,β-butyrolactone moiety. Ibrahim et al. (2026) reviewed 238 cassane-type diterpenoids with diverse bioactivities, including antiviral, anticancer, anti-inflammatory, antimalarial, and antiproliferative effects. Cleistanthanes (242-254, 265-270) are defined by an aromatized C-ring as their core structural feature, and have been documented in 201 distinct compounds (Yang et al., 2025).

Tetracyclic diterpenoids (290-299) generally feature a 6/6/6/5 ring system and are classified based on their configuration into those with a bridged ring at C-8 (i.e., *ent*-kaurane and *ent*-gibberellane types) or at C-9 (Zhu et al., 2022). *Ent*-kaurane-type diterpenoids (290-297) are the enantiomers of kaurane diterpenoids and were first discovered in the leaf oil of *Agathis australis* (Tu et al., 2023). Among this class, the most extensively studied compounds are steviol (290) (Pelczynska et al., 2025), utilized as a sweetener, and oridonin (294), which possesses potent anticancer and anti-inflammatory activities (Han et al., 2025; Shi et al., 2026).

Macrocyclic diterpenoids (300-406) are a class of diterpenoids that contain at least one seven-membered or larger carbocyclic ring in their structure. They are typically highly oxygenated and possess complex structural features. Macrocyclic diterpenoids are structurally diverse and encompass several major types, including casbene, jatrophanes, lathyranes, cephalotane, daphnane, grayanane, tigliane, and crotofolane. Casbene-type diterpenoids (300-303) feature a 14-membered macrocyclic ring and are currently regarded as important biosynthetic precursors for other macrocyclic diterpenoids. Jatrophanes (304-307, 309-315) and lathyranes (308, 316-318) contain macrocyclic rings of ten or more carbon atoms. Due to their structural instability, they are relatively rare in nature and have only been reported in plants of the Euphorbiaceae family (Chen et al., 2025a). Cephalotane diterpenoids (366-384) possess a rigid 7/6/5/6 fused tetracyclic structure and are exclusively found in the genus *Cephalotaxus*, with 105 structurally diverse compounds identified to date (Li et al., 2023a). Crotofolanes (385-391) feature a 5/6/7 tricyclic ring system and are primarily reported in the genus Croton (Euphorbiaceae). Zhao et al. (2025b) documented 62 crotofolane derivatives isolated from 11 Croton species. Tiglianes (321-337, 338-341, 353) are based on a 5/7/6/3 tetracyclic system, characterized by an α,β-unsaturated ketone structure in the A-ring. Tiglianes typically bear multiple hydroxy groups at C-12, C-13, and C-20, which can be readily esterified with acetic, isobutyric, crotonic, 2-methylbutyric, benzoic, 2-methylaminobenzoic acid, or saturated and unsaturated long-chain fatty acids (Wang et al., 2015; Jiang et al., 2022). The distribution of tigliane diterpenoids is restricted to plant species of the Euphorbiaceae and Thymelaeaceae families (Zhao et al., 2025b). Grayanane diterpenoids (354-365) are particularly notable. These terpenes are characterized by a unique 5/7/6/5 tetracyclic system and are exclusive to the Ericaceae family (Niu et al., 2018; Li et al., 2020). They represent an important class of compounds involved in plant defense against herbivorous insects, with over 250 grayanane diterpenoids reported to date (Liu et al., 2024). Daphnane diterpenoids (342, 343, 349-352), as one of the representative types of diterpenoid compounds with rich structural diversity and significant biological activities, possess a 5/7/6 tricyclic skeleton mainly found in species of the Thymelaeaceae and Euphorbiaceae families (Xie et al., 2021). Currently, over 300 daphnane diterpenoids have been reported (Ding et al., 2025). In addition, paclitaxel is a diterpenoid compound with a complex structure, featuring a 6/8/6/4 tetracyclic carbon skeleton that contains up to 11 stereocenters and exhibits a high degree of oxidation. Paclitaxel was originally isolated from *Taxus brevifolia* and possesses remarkable anticancer activity, making it an effective treatment for a broad spectrum of cancers (Bergman et al., 2019).

### 2.2 Distribution in medicinal plants

**Figure 2:**
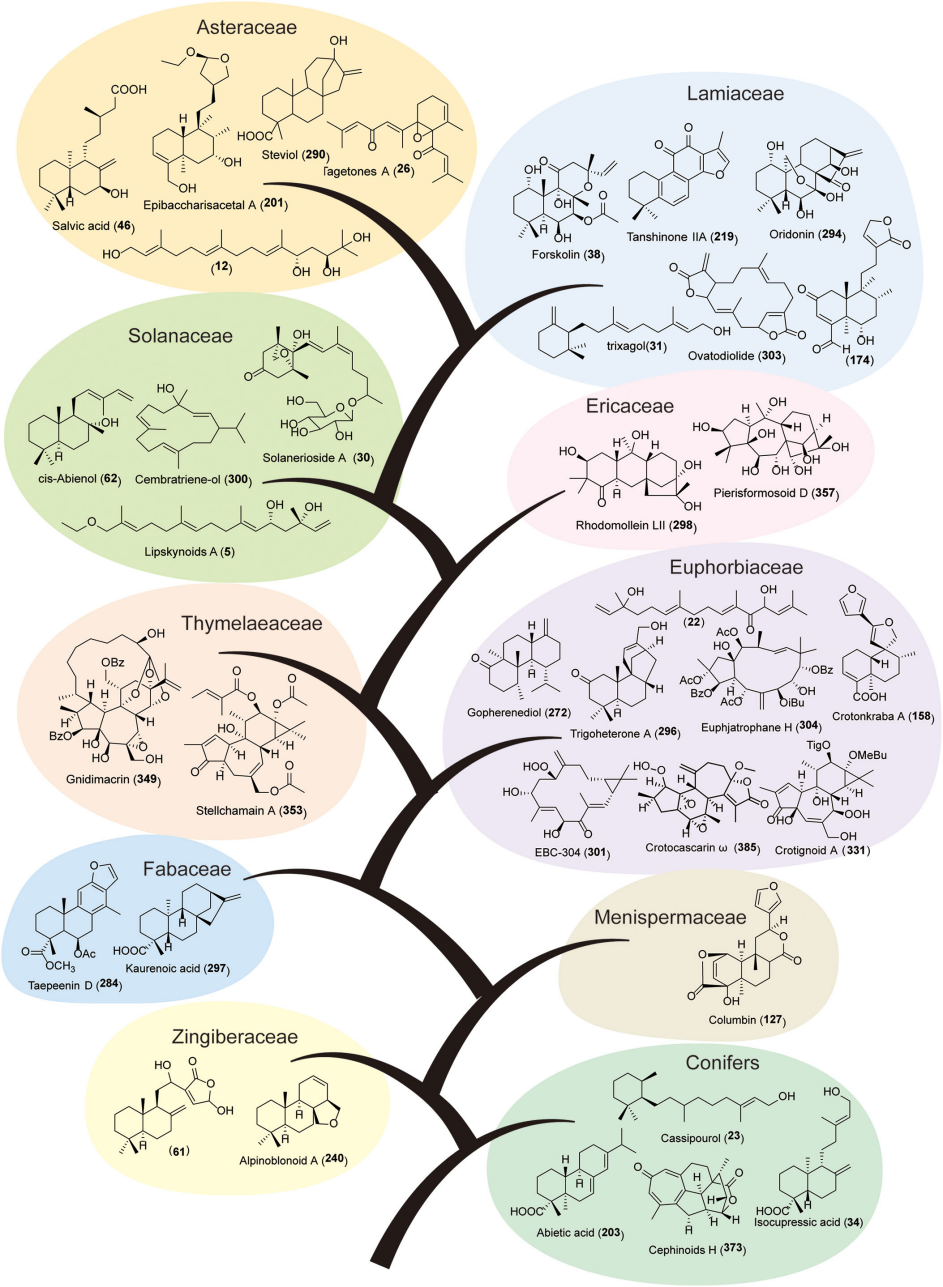
Main distributions of diterpenoids in nature.

Diterpenoids are widely distributed and structurally diverse in nature, occurring in fungi, algae, monocotyledons, dicotyledons, and marine organisms. In medicinal plants, diterpenoids are primarily distributed in conifers, Zingiberaceae, Fabaceae, Asteraceae, Oleaceae, Ericaceae, Acanthaceae, Euphorbiaceae, Verbenaceae, Lamiaceae, and Solanaceae (**Figure 2**).

Conifers harbor multiple diterpenoid types. For instance, monocyclic diterpenoid cassipourol (22) has been isolated from *Dacrycarpus imbricatus* (Tam et al., 2019), labdane-type cuceolatins A–C (31-33) from *Cunninghamia lanceolata* (Yu et al., 2019), tricyclic abietane-type abietic acid (203) from *Pinus palustris* (Ito et al., 2020; Ren et al., 2026). In monocotyledonous Zingiberaceae, bicyclic and tricyclic diterpenoids have been reported, including alpinoblonoids A and B (240, 241) with a unique tricyclic structure from *Alpinia oblongifolia* (Zhang et al., 2025a). In the Menispermaceae family, only bicyclic clerodane-type diterpenoids have been reported from *Tinospora capillipes* and *T. sinensis*, similarly, only clerodanes have been found in *Casearia graveolens* (Salicaceae). Fabaceae plants produce tricyclic and tetracyclic diterpenoids, including the cassane-type diterpenoids taepeenin D (284), caeminaxins A and B (282, 283), which are characteristic of this family (Lu et al., 2023; Wang and Wang, 2025).

The Euphorbiaceae family exhibits the greatest diversity of diterpenoids, encompassing nearly all structural types, and serves as a key subject for investigating macrocyclic diterpenoid diversity (Wang et al., 2015; Zhao et al., 2025b). Researchers have isolated labdanes, halimanes, clerodanes, abietanes, cleistanthanes, and *ent*-kauranes from species such as *Croton sublyratus*, *C. laevigatus*, *C. caudatus*, *C. floribundus*, *C. krabas*, *C. insularis*, *Suregada zanzibariensis*, *Euphorbia fischeriana*, *E. helioscopia*, and *Strophioblachia fimbricalyx*. Despite the limited number of diterpenoid reports from the Thymelaeaceae family, several tetracyclic diterpenoids have been characterized. Representative compounds include gnidimacrin and daphneodorins A–C (349-352) isolated from *Daphne odora* (Otsuki et al., 2020), along with stellchamain A (353) from *Stellera chamaejasme* (Nie et al., 2025). From the Ericaceae family, tetracyclic and macrocyclic diterpenoids with unique structures have been isolated. For example, rhodomollein LII and LIII (298, 299) from *Rhododendron molle* are leucothane-type tetracyclic diterpenoids (Li et al., 2020), while pierisformosoids A–L (354-365) from *Pieris formosa* belong to the grayanane type, featuring a 5/7/6/5 ring system (Niu et al., 2018). The Solanaceae family exhibits diverse diterpenoid types. Acyclic diterpenoid derivative capsianosides (3-5) have been isolated from *Capsicum* spp (Chilczuk et al., 2020), monocyclic diterpenoid solanerioside A (27) from *Solanum erianthum* (Peng et al., 2017), and labdane-type diterpenoid *cis*-abienol (62) along with macrocyclic diterpenoid cembratriene-ol (300) from *Nicotiana* spp (Qi et al., 2021; Xu et al., 2024). Notably, no tricyclic or tetracyclic diterpenoids have been reported in Solanaceae to date, suggesting that this family may lack kaurene synthase-like (KSL) enzymes functional in *ent*-kaurane biosynthesis.

Many medicinal plants in the Lamiaceae family are rich in diterpenoids, including the monocyclic diterpenoid trixagol (28) and bicyclic diterpenoids from *Rosmarinus officinalis* (Xu et al., 2025), the macrocyclic diterpenoid ovatodiolide (303) from *Anisomeles indica* (Zhou et al., 2026), the tricyclic diterpenoids tanshinone I (218), tanshinone IIA (219), and cryptotanshinone (220) from *Salvia miltiorrhiza*, and the tetracyclic diterpenoid oridonin (294) from *Rabdosia rubescens* (Li et al., 2025b). Furthermore, numerous bicyclic diterpenoids have been isolated from families within the Lamiales order, including Oleaceae, Verbenaceae, Acanthaceae, and Scrophulariaceae. Examples include scoparicol E (45) from *Scoparia dulcis* (Dong et al., 2024), calintegerinoids A–E (182–186) from *Callicarpa integerrima* (Zeb et al., 2025), forsyditerpenes A–L (187-198) from *Forsythia suspensa* (Li et al., 2025a), and andrographolide (35) from *Andrographis paniculata* (Tundis et al., 2023).

The Asteraceae, one of the largest plant families, also exhibits remarkable diterpenoid diversity. For example, acyclic diterpenoid lipskynoids A–G (6-12) have been isolated from *Carpesium lipskyi* (Zhong et al., 2022), monocyclic diterpenoid tagetones A and B (25, 26) from *Tagetes minuta* (Ibrahim and Mohamed, 2017), labdane-type salvic acid (46) from *Eupatorium salvia* (Echeverria et al., 2017), clerodane-type baccharisacetals A and B (199, 200) from *Baccharis trimera* (Liang et al., 2026), and the tetracyclic diterpenoid steviol (290) from *Stevia rebaudiana* (Pelczynska et al., 2025).

Beyond the aforementioned families, diterpenoids have been reported in several other plant families, such as Saururaceae, Phyllanthaceae, Meliaceae, Potamogetonaceae, Caesalpiniaceae, Velloziaceae, Simaroubaceae, Annonaceae, and Icacinaceae. Collectively, the distribution of diterpenoids exhibits certain taxonomic regularities, with more evolutionarily advanced groups tending to produce structurally more complex compounds, while certain lineages possess unique types.

## 3 Biological activity of diterpenoids

### 3.1 Defense-related responses in plants

Diterpenoids play a crucial role in plant innate immunity, functioning as phytoalexins or signaling molecules to defend against pathogens and herbivores through diverse mechanisms (**Table 1**). For instance, capsianosides (3-5) are specifically involved in defense-related responses to biotic stress (Chilczuk et al., 2020). *cis*-abienol (62) has demonstrated practical utility by inducing resistance in tomato against bacterial wilt caused by *Ralstonia solanacearum*, a pathogen that severely compromises tomato yield and quality (Zhang et al., 2025b). Geranyllinalool (2) is considered to possess insecticidal properties, acting as a toxic deterrent or repellent against herbivorous insects (Su et al., 2017; Liu et al., 2018a). Cembratriene-ol (300) exhibits both antimicrobial and insecticidal activities, specifically noted for its ability to disrupt fungal endomembrane structures, with a concentration of 80 μg/mL completely inhibiting the growth of the plant pathogen *Valsa mali* (Xu et al., 2024). Pierisformosoids A, D and I (354, 357, 362) display antifeedant activity against the major agricultural pest *Plutella xylostella* at a concentration of 0.5 mg/mL (Niu et al., 2018). Furthermore, tigliane-type diterpenoids (328-330) exhibit potent larvicidal activity against *P. xylostella*, with LC_50_ values of 0.19, 0.16, and 0.26 μM, respectively, which are comparable to that of the positive control flubendiamide (LC_50_ = 0.14 μM) (Jiang et al., 2022). These studies highlight the potential of diterpenoids to provide valuable leads for the development of green pesticides and resistance inducers.

### 3.2 Anti-inflammatory

The search for novel anti-inflammatory agents remains challenging due to the complexity of inflammatory processes and their dual role in host defense. Over the past few decades, however, accumulating evidence has demonstrated that diterpenoids, particularly bicyclic diterpenoids, play a significant role in the therapeutic intervention of various inflammatory diseases (**Table 1**). Numerous diterpenoids have demonstrated the ability to inhibit nitric oxide (NO) production in LPS-stimulated RAW 264.7 macrophages, a key indicator of anti-inflammatory activity mediated through modulation of the NF-κB pathway (Tran et al., 2017). For instance, lipskynoids A–G (6–12) and the bicyclic diterpenoid 86 exhibited potent activity, with IC_50_ values ranging from 9.9 to 18.47 μM (Zhong et al., 2022; Qi et al., 2024). Beyond the inhibition of NO production, some diterpenoids target more specific inflammatory pathways. For instance, calintegerinoid C (184) and euphzycopins D (320) blocks NLRP3 inflammasome activation, with IC_50_ values of 1.27 μM for lactate dehydrogenase (LDH) release inhibition and 7.75 μM, respectively (Qiu et al., 2024b; Zeb et al., 2025). These findings confirm the NLRP3 inflammasome as a key molecular target for diterpenoid-based anti-inflammatory therapy. Among the most thoroughly studied diterpenoids with respect to anti-inflammatory mechanisms is andrographolide (35). Andrographolide has been reported to show therapeutic potential in multiple inflammatory conditions, including asthma, rheumatoid arthritis, inflammation-mediated neurodegenerative diseases, autoimmune encephalomyelitis, and systemic lupus erythematosus (Hu et al., 2024; Messire et al., 2024). Mechanistic studies have revealed that it forms a covalent adduct with the reduced Cys62 residue on the p50 subunit through Michael addition involving its △12(13)-exocyclic double bond, thereby blocking NF-κB binding to its consensus sequence and attenuating its transcriptional activity (Tundis et al., 2023; Messire et al., 2024). Currently, several andrographolide-containing proprietary Chinese medicines have been approved for clinical use, including Kan Jang tablets, andrographolide tablets, andrographolide drop pills, and andrographolide capsules (Loureiro Damasceno et al., 2022).

### 3.3 Anticancer

A vast number of diterpenoids exhibit cytotoxic effects against a broad panel of human cancer cell lines, with some demonstrating notable selectivity and potency (**Table 1**). For instance, tagetone A (25) displays potent cytotoxicity against MCF-7 (breast) and A549 (lung) cancer cells, with IC_50_ values of 4.68 and 4.24 μM, respectively, while tagetone B (26) is selectively active against HCT116 (colon) cancer cells (IC_50_ = 6.30 μM) (Ibrahim and Mohamed, 2017). Isocupressic acid (34) demonstrates superior potency against HepG2 (liver) cancer cells (IC_50_ = 3.73 μg/mL) compared to the standard chemotherapeutic drug cisplatin (IC_50_ = 12.65 μg/mL) (Naderian et al., 2025). Crotonmekongenin A (295) shows significant cytotoxic activity against FaDu (pharyngeal squamous carcinoma), HT-29 (colorectal adenocarcinoma), and SH-SY5Y (neuroblastoma) cell lines, with ED_50_ values of 0.48, 0.63, and 0.45 μg/mL, respectively (Udomthawee et al., 2021). Additionally, the macrocyclic crotignoid A (331) displays potent activity against HL-60 (leukemia) cells, with an IC_50_ value of 1.61 μM (Zhang et al., 2015). Beyond *in vitro* cytotoxicity studies, the *in vivo* anticancer mechanisms of several diterpenoids have been intensively investigated. Ovatodiolide (303) exerts its anticancer effects through modulation of key signaling pathways, including NF-κB/MMP-9, JAK2/STAT3, PI3K/AKT/mTOR, and Wnt/β-catenin, and effectively targets cancer stem cells (Zhou et al., 2026). Furthermore, *in vivo* evidence for the therapeutic potential of diterpenoids is provided by compound 151, which effectively inhibits angiogenesis and displays significant anticancer activity in *zebrafish xenograft* models (Wang et al., 2024a). It is worth noting that the bioactivity and toxicity of diterpenoids are often closely associated, which has limited their clinical application. Therefore, reducing their toxicity is an urgent issue that needs to be addressed.

### 3.4 Antimicrobial

Diterpenoids have demonstrated considerable promise as antimicrobial agents against a broad spectrum of bacterial, fungal, and viral pathogens, including clinically relevant drug-resistant strains (**Table 1**). Notable examples with antibacterial activity include abietic acid (203), preparations of which are applied to various skin injuries and infections, including wounds, bites, burns, paronychia, impetigo (with *Staphylococcus aureus* as a major pathogen), and skin fissures. Furthermore, abietic acid inhibits the growth of the cariogenic bacterium *S. mutans* and is used as a key component in dental filling materials (Kameyama et al., 2020). Additionally, cleistanthol (242) exhibits broad-spectrum activity against various Gram-positive bacteria and *Shigella flexneri*, with MIC_50_ values ranging from 12 to 64 μg/mL (Çiçek et al., 2020). Labdane-type diterpenoid 42 shows potent and selective inhibition against *Bacillus subtilis* and *P. aeruginosa* with MIC values as low as 0.125 and 1 μg/mL, respectively (Xu et al., 2025). In terms of antiplasmodial activity, mangiolide (227) stands out for its remarkable broad-spectrum effects, demonstrating potent activity against both chloroquine-sensitive and -resistant *Plasmodium falciparum* strains (IC_50_ ~ 0.8 μg/mL), along with activity against *Cryptococcus neoformans*, methicillin-resistant *S. aureus* (MRSA, IC_50_ = 3.90 μg/mL), and vancomycin-resistant *Enterococcus* (VRE, IC_50_ = 7.20 μg/mL) (Matundura et al., 2023). In the antiviral arena, gnidimacrin (349) and daphneodorins A–C (350-352) potently inhibit HIV-1 replication at subnanomolar concentrations, with EC_50_ values as low as 0.061 nM for gnidimacrin (Otsuki et al., 2020). Crotokilwaepoxides B and E (387, 390) exhibit modest antiviral activity against human rhinovirus type 2 (HRV-2) (Mahambo et al., 2023), while dehydroabietic acid protects against foot-and-mouth disease virus (FMDV) by targeting the viral RNA-dependent RNA polymerase (3Dpol) (Mana et al., 2024). Collectively, the remarkable structural and functional diversity of these diterpenoids—particularly their activity against WHO priority pathogens such as MRSA, VRE, and *P. aeruginosa*—highlights their potential as lead compounds for antimicrobial drug development.

### 3.5 Other biological activities

Beyond these well-established activities, diterpenoids exhibit a wide range of other pharmacological effects (**Table 1**). In the context of metabolic disorders, several diterpenoids have demonstrated promising antidiabetic effects through distinct mechanisms. At the cellular level, compounds 13 and 14 increase Nrf-2/HO-1 levels while decreasing NF-κB, TNF-α, and IL-6 in glomerular mesangial cells (GMCs) at 30 μM, suggesting potential for mitigating diabetic complications (Chen et al., 2022). Tinosinoids G and H (140, 141) inhibit α-glucosidase with IC_50_ values of 46.0 and 29.7 μM, respectively, providing a mechanism for postprandial blood glucose control (Zhang et al., 2022). Regarding adipocyte function, lauenones A and B (92, 93) suppress adipogenesis in 3T3-L1 cells (EC_50_ = 13.07–42.57 μM) more effectively than metformin, with their stereochemistry modulating the PPARγ/C/EBPα pathway (Zhang et al., 2025c). *In vivo* evidence further supports these findings. Scoparicol E (45) exhibits direct pancreatic activity by reducing MLD-STZ-induced hyperglycemia in mice and increasing insulin levels and modulating islet apoptosis (Dong et al., 2024). At the systemic level, steviol (290), a naturally occurring diterpenoid sweetener, demonstrates broader metabolic benefits by lowering cholesterol, triglycerides, LDL, and HbA1c—a key marker of long-term glucose control (Pelczynska et al., 2025).

In addition to these metabolic effects, diterpenoids exhibit notable activities targeting the nervous system, circulatory system, and liver protection. In the context of neurodegenerative diseases, euphorfinoid E (232) inhibits acetylcholinesterase (AChE) with an IC_50_ value of 6.23 μM (Wei et al., 2021). Several tetracyclic and grayanane-type macrocyclic diterpenoids from Ericaceae plants demonstrate potent analgesic effects *in vivo*. Notably, rhodomollein LIII (300) displays significant antinociceptive activity in an acetic acid-induced writhing test in mice, achieving an inhibition rate of 89.0% at 20 mg/kg (Li et al., 2020), while pierisformosoids (354, 355, 357, 358, 360, 361) show marked activity at a lower dosage of 5.0 mg/kg (Niu et al., 2018). For the circulatory system, the sulfonated sodium salt of tanshinone IIA has been approved in China for the treatment of cardiovascular disease since the 1980s. Accumulating evidence suggests that tanshinone IIA attenuates maladaptive vascular smooth muscle cell (VSMC) behaviors by modulating calcium signaling, regulating programmed cell death pathways, and inhibiting pro-inflammatory signaling cascades (Li et al., 2025b). Additionally, crassifolins Q–U (114-118) exhibit anti-angiogenic effects by reducing vascular network formation through modulation of VEGF signaling, with crassifolin U (118) showing the most potent activity, with IC_50_ values ranging from 7.20 to 48.27 μM (Li et al., 2021). Additionally, baccharisacetals A and B (199, 200) and their epimers show hepatoprotective effects against alcohol-induced hepatic injury in both LX-2 cells and zebrafish models (Liang et al., 2026).

## 4 Biosynthetic pathways of diterpenoids

**Figure 3:**
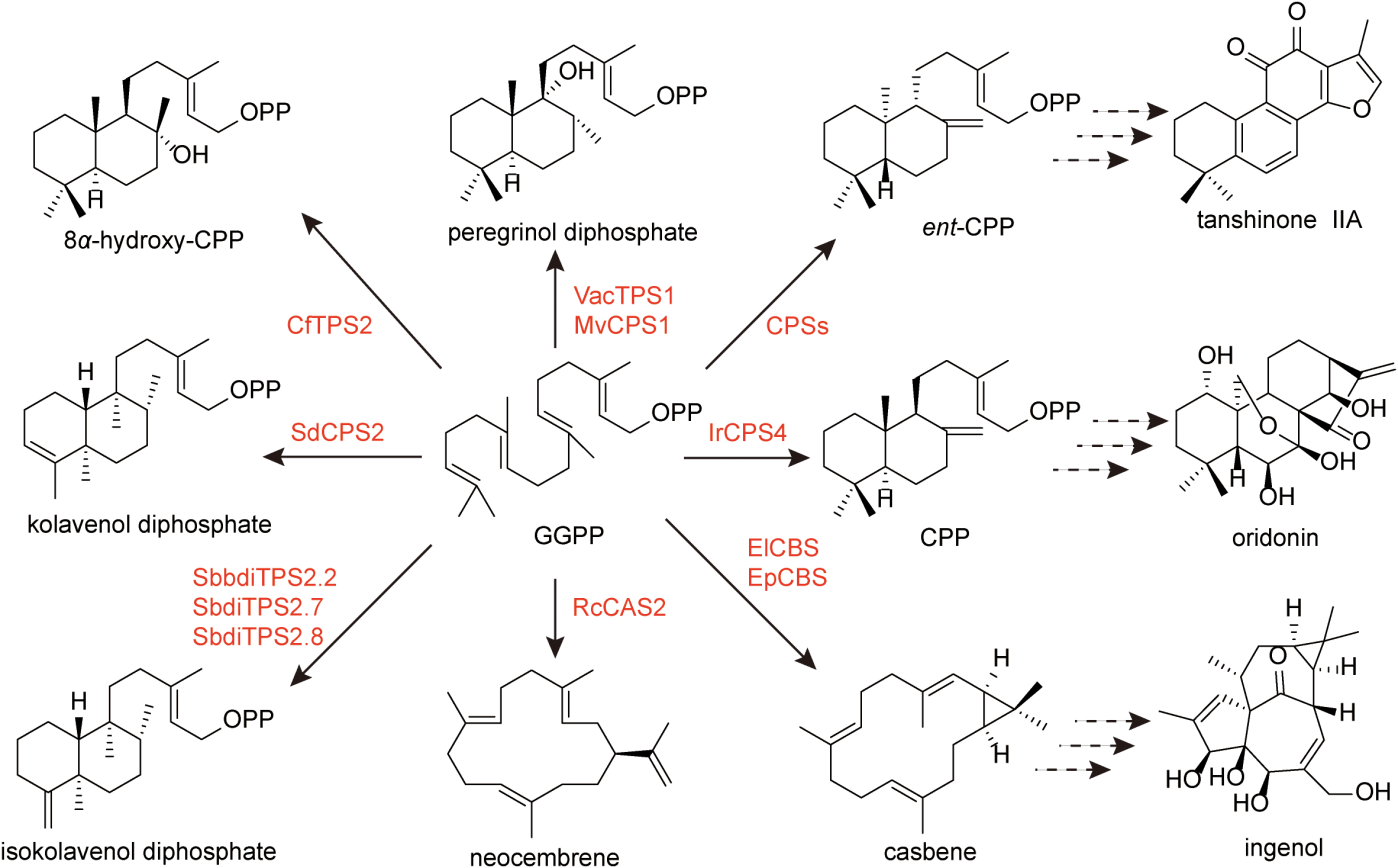
The biosynthesis of skeleton structures of diterpenoids.

As described above, diterpenoids exhibit remarkable structural diversity, ranging from bicyclic to tetracyclic and macrocyclic skeletons. However, regardless of their structural complexity, their biosynthesis invariably originates from the universal precursor GGPP (**Figure 3**), which is primarily generated through the MEP pathway in plants. These pathways typically initiate with the action of class II and class I diTPSs, followed by oxidative modifications catalyzed by CYP450s and various tailoring enzymes (Cao et al., 2010; Frey et al., 2025). With the rapid advances in genome and transcriptome sequencing, an increasing number of biosynthetic steps have been unraveled, offering valuable opportunities to enhance diterpenoid production or enable their heterologous biosynthesis.

### 4.1 Biosynthesis of labdane-type diterpenoids

**Figure 4:**
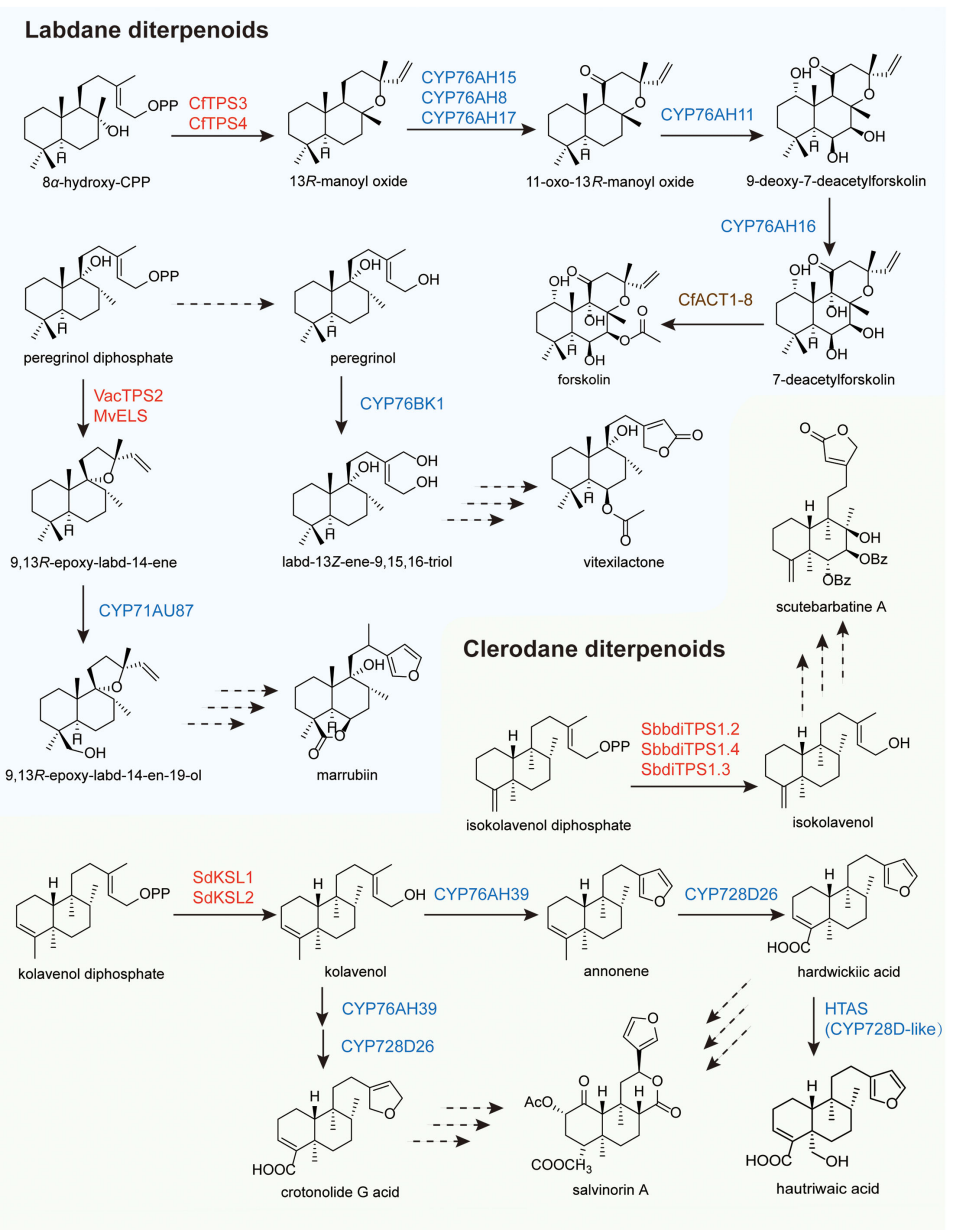
Biosynthetic pathways of bicyclic diterpenoids.

The biosynthesis of labdane-type diterpenoids has been characterized in three medicinal plants (*Coleus forskohlii*, *Marrubium vulgare*, and *Vitex agnus-castus*) revealing both conserved enzymatic machinery and pathway-specific diversification (**Figure 4**).

In *C. forskohlii*, the forskolin biosynthetic pathway represents the most completely elucidated labdane-type diterpenoid pathway to date (Hu et al., 2021). Beginning with GGPP, the class II diTPS CfTPS2 catalyzes cyclization to form 8*α*-hydroxy-copalyl diphosphate (8*α*-hydroxy-CPP), which is then stereospecifically converted to 13*R*-manoyl oxide by the class I diTPSs CfTPS3/4 (Pateraki et al., 2014). Oxidation at C-11 is independently catalyzed by three CYP76AH subfamily enzymes (CfCYP76AH15, CfCYP76AH8, and CfCYP76AH17) with CfCYP76AH15 demonstrating the greatest efficiency and specificity. CfCYP76AH11 catalyzes oxygenations at C-1, C-6, and C-7 to produce 9-deoxy-7-deacetylforskolin, CfCYP76AH16 mediates C-9 oxygenation to yield 7-deacetylforskolin, and CfACT1–8 completes the pathway via C-7 acetylation (Pateraki et al., 2017).

In contrast to the fully elucidated forskolin pathway, the biosynthesis of marrubiin in *M. vulgare* has only been partially characterized. The pathway is initiated by the class II diTPS MvCPS1, which converts GGPP to peregrinol diphosphate, a distinct intermediate from the 8*α*-hydroxy-CPP found in forskolin biosynthesis (Zerbe et al., 2014). This intermediate is subsequently cyclized by the class I diTPSs MvELS to form 9,13-epoxy-labd-14-ene (Zerbe et al., 2014). Further modification is catalyzed by the CYP450 enzyme CYP71AU87, which produces two isomeric hydroxylated products: 9,13-epoxy-labd-14-en-18-ol and 9,13-epoxy-labd-14-en-19-ol (Karunanithi et al., 2019). These isomers are proposed as key intermediates en route to marrubiin. However, the subsequent steps, which include furan ring formation and additional oxidations required to complete the marrubiin structure, remain to be elucidated.

A parallel situation is observed in *V. agnus-castus*, where the biosynthetic pathway leading to vitexilactone shares early steps with marrubiin biosynthesis but diverges at later stages. The enzymes VacTPS1 and VacTPS2 catalyze the formation of peregrinol diphosphate and 9,13-epoxy-labd-14-ene, respectively—identical to the corresponding steps in *M. vulgare* (Heskes et al., 2018). Peregrinol diphosphate is presumed to be converted to peregrinol by an uncharacterized diTPSs. Subsequently, peregrinol undergoes oxidation by CYP76BK1 to generate labd-13Z-ene-9,15,16-triol (Heskes et al., 2018). This trihydroxylated intermediate is hypothesized to be the precursor of vitexilactone. The conversion of this triol to vitexilactone likely involves additional oxidation steps, lactone ring formation, and possibly further tailoring modifications, all of which await discovery.

### 4.2 Biosynthesis of clerodane-type diterpenoids

Clerodane diterpenoids, though less extensively studied than their labdane-type counterparts and lacking a fully elucidated biosynthetic pathway, have nonetheless yielded notable findings (**Figure 4**). A significant discovery is the identification of a CYP450 involved in furan ring formation, a key modification in the biosynthesis of salvinorin A that holds considerable importance for structural diversification.

In *Salvia divinorum*, the biosynthesis of salvinorin A is initiated by the class II diTPS SdCPS2, which converts GGPP to kolavenyl diphosphate (KPP) (Chen et al., 2017). Subsequently, the class I diTPSs SdKSL1 and SdKSL2 catalyze the formation of kolavenol from KPP (Pelot et al., 2017). Interestingly, heterologous expression of SdCPS2 alone in tobacco and yeast unexpectedly yielded kolavenol, suggesting that endogenous phosphatases in these hosts can cleave the diphosphate group (Pelot et al., 2017). Following this, CYP76AH39 catalyzes the conversion of kolavenol to crotonolide G, a dihydrofuran-containing clerodane (Kwon et al., 2022). Notably, a homologous enzyme from *S. splendens* produces annonene, a furan-containing clerodane, indicating functional divergence within the CYP76AH subfamily. Further downstream, CYP728D26 catalyzes three successive oxidations at the C-18 position of both crotonolide G and annonene, yielding crotonolide G acid and hardwickiic acid, respectively (Ngo et al., 2024). These intermediates represent the fully oxidized backbones of salvidivin A and salvinorin A. In a recent advancement, a novel enzyme, hautriwaic acid synthase (HTAS, CYP728D-like), was identified in Salvia splendens and shown to be responsible for the biosynthesis of hautriwaic acid, providing new insights into clerodane diversification (Lin et al., 2025a).

Scutebarbatine A, a highly modified clerodane diterpenoid isolated from *Scutellaria barbata*, represents a structural class distinct from that of salvinorin A. Its biosynthesis has been investigated in both *S. barbata* and *S. baicalensis* (Li et al., 2023b). The pathway is initiated by the conversion of GGPP to isokolavenyl diphosphate (IKPP), catalyzed by the class II diTPSs SbbdiTPS2.3 (*S. barbata*), and SbdiTPS2.7 and SbdiTPS2.8 (*S. baicalensis*). IKPP is subsequently converted to isokolavenol by class I diTPSs, including SbbdiTPS1.2 and SbbdiTPS1.4 in *S. barbata*, and SbdiTPS1.3 in *S. baicalensis*. The transformation of isokolavenol into scutebarbatine A involves extensive structural modifications, including multiple hydroxylations, esterifications with side chains, and furan ring formation. Although four P450-catalyzed hydroxylation steps have been predicted, efforts to identify P450 enzymes capable of accepting isokolavenol as a substrate have so far been unsuccessful, leaving the downstream portion of the pathway unresolved.

The biosynthesis of clerodane diterpenoids remains a topic of significant interest. In a recent study, homologs of VacCYP76BK1 from ten different species, including *Ajuga reptans* and *Callicarpa americana*, were cloned and transiently expressed in *Nicotiana benthamiana* together with the upstream synthases CamTPS2 or ArTPS2 (Johnson et al., 2019; Hamilton et al., 2020). Functional assays revealed that these CYP76BK1 enzymes catalyze the oxidative cyclization of clerodane backbones to yield a furan ring. This discovery advances our understanding of furan ring assembly in clerodane diversification (Schlecht et al., 2024). Further exploration of P450 functionality and substrate specificity, combined with synthetic biology approaches, may ultimately enable the complete reconstruction of salvinorin A and related biosynthetic pathways in heterologous hosts.

### 4.3 Biosynthesis of tricyclic diterpenoids

**Figure 5:**
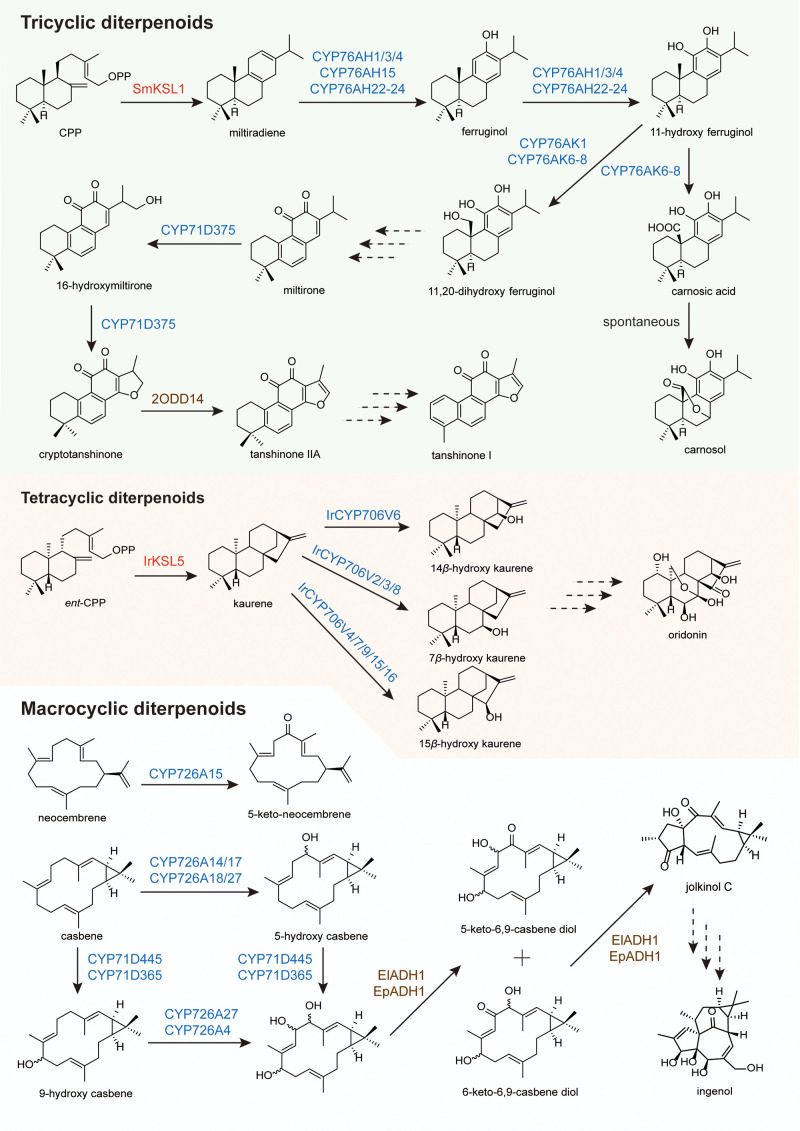
Biosynthetic pathways of tricyclic, tetracyclic, and macrocyclic diterpenoids.

Biosynthetic investigations of tricyclic abietane diterpenoids have been primarily conducted in plants of the Lamiaceae family (**Figure 5**). The pathway is initiated by the cyclization of GGPP to copalyl diphosphate (CPP), a reaction catalyzed by class II diTPSs. In *Salvia miltiorrhiza*, SmCPS1 and SmCPS2 perform this conversion, and the resulting CPP is subsequently cyclized by the class I diTP SmKSL1 to yield miltiradiene (Gao et al., 2009; Brückner et al., 2014). This olefin serves as the universal hydrocarbon backbone for diverse abietane diterpenoids and is believed to undergo spontaneous oxidation to abietatriene under physiological conditions (Zi and Peters, 2013).

The oxidative functionalization of this tricyclic scaffold is primarily orchestrated by CYP450s belonging to the CYP76 and CYP71 clans. Many of these enzymes exhibit notable substrate promiscuity, a feature that contributes significantly to the structural diversity characteristic of abietane-producing plants. The initial oxygenation step is catalyzed by ferruginol synthase (CYP76AH1 in *S. miltiorrhiza*), which introduces a hydroxyl group at C12 to produce ferruginol (Guo et al., 2013). In *Rosmarinus officinalis* and various sage species, homologous enzymes (CYP76AH22–24) catalyze sequential oxidations, yielding 11-hydroxyferruginol bearing hydroxyl groups at both C11 and C12 (Ignea et al., 2016a; Scheler et al., 2016). At this juncture, the pathway bifurcates into two distinct branches leading to tanshinones and carnosic acid/carnosol, respectively.

In the tanshinone branch, represented by *S. miltiorrhiza*, 11-hydroxyferruginol undergoes oxidation at C20 catalyzed by CYP76AK1, producing 11,20-dihydroxyferruginol (Guo et al., 2016). This intermediate then proceeds through several incompletely resolved steps, including demethylation at C20 and aromatization of the B ring, to form miltirone. Miltirone subsequently serves as the substrate for D-ring formation, in which CYP71D375 and CYP71D373 first hydroxylate miltirone at C16 and then catalyze heterocyclization with C14 to generate the characteristic furan D-ring through a 14,16-ether linkage, yielding cryptotanshinone (Ma et al., 2021). Cryptotanshinone is reduced by the 2-oxoglutarate-dependent dioxygenase Sm2ODD14 to produce tanshinone IIA (Song et al., 2022). Hydroxylation at C19, potentially mediated by CYP71D373, converts tanshinone IIA to tanshinone IIB (Qiu et al., 2024a). The biosynthesis of tanshinone I requires demethylation at C4 with concomitant aromatization of ring A, with oxidation at C18 or C19 representing likely intermediate steps. Although genomic and transcriptomic data have implicated candidate genes in these final transformations, the complete tanshinone pathway remains to be fully elucidated.

In parallel, the carnosic acid branch operating in *R. officinalis* and sage employs CYP76AK6–8 to catalyze three successive oxidations at C20 of 11-hydroxyferruginol, converting the hydroxymethyl group sequentially to an aldehyde and then to a carboxylic acid, thereby generating carnosic acid (Ignea et al., 2016a; Scheler et al., 2016). This carboxylation is a hallmark of the carnosic acid pathway and does not occur in tanshinone biosynthesis. Carnosic acid spontaneously undergoes oxidation to carnosol, a major constituent of rosemary extracts. In *S. pomifera*, an alternative oxygenation pattern is observed, in which CYP71BE52 hydroxylates ferruginol at the 2α position to produce salviol, further illustrating the catalytic versatility of P450 enzymes in this family.

### 4.4 Biosynthesis of tetracyclic diterpenoids

Oridonin from *Isodon rubescens* represents one of the more extensively studied tetracyclic diterpenoids. Its biosynthesis is initiated by the conversion of GGPP to ent-copalyl diphosphate (*ent*-CPP), catalyzed by IrCPS4 (**Figure 5**) (Jin et al., 2017). The class I diTPS IrKSL5 subsequently cyclizes *ent*-CPP to *ent*-kaurene, establishing the tetracyclic scaffold (Jin et al., 2017). From this intermediate, the construction of oridonin requires oxidation at six distinct positions. A biosynthetic gene cluster on chromosome 2, comprising tandem-duplicated CYP706V oxidase genes, has been identified (Sun et al., 2023). Functional characterization revealed specialization within this family: three enzymes (IrCYP706V2, V3, V8) independently convert ent-kaurene to 7β-hydroxykaurene, while five enzymes (IrCYP706V4, V7, V9, V15, V16) produce 15β-hydroxykaurene. A single P450, IrCYP706V6, specifically catalyzes 14β-hydroxylation to yield 14β-hydroxykaurene. Although these hydroxylation events are all required for oridonin biosynthesis, the sequential order in which they occur remains to be determined.

Beyond the oxidative modifications that generate the oxygenated scaffold, glycosylation constitutes another key layer of structural diversification in diterpenoid metabolism. The stepwise glycosylation catalyzed by UGTs has been largely elucidated through studies on steviol glycoside biosynthesis. In *Stevia rebaudiana*, the pathway begins with *ent*-kaurenoic acid, which is hydroxylated at C13 by kaurenoic acid 13-hydroxylase (SrKA13H) to generate the aglycone steviol (Wang et al., 2016b). SrUGT85C2 initiates the process by glycosylating the C13 hydroxyl group to produce steviolmonoside. Subsequent glucosylation at the C2’ hydroxyl of the C13-glucose moiety is catalyzed by SrUGT91D2, forming steviolbioside. SrUGT74G1 then adds a glucose at the C19 carboxyl group to yield stevioside, and finally, SrUGT76G1 catalyzes glucosylation at the C3’ hydroxyl of the C13-glucose, producing rebaudioside A (Wang et al., 2016b). Beyond the stevia pathway, rubusoside, another steviol glycoside found in *Rubus suavissimus* and *Angelica keiskei*, has been characterized for its ability to inhibit fructose and glucose transporters. Comparative transcriptomics of *R. suavissimus* and *A. keiskei* has identified six UGTs that expand the known glycosylation repertoire: RsUGT75L20, RsUGT75T4, AkUGT75L21, and AkUGT75W2 catalyze C19 glycosylation of ent-kaurenoic acid, steviol, and steviolmonoside, while RsUGT85A57 and AkUGT85A58 function as 13-O-glycosyltransferases that convert steviol 19-O-glucoside to rubusoside (Sun et al., 2018).

### 4.5 Biosynthesis of macrocyclic diterpenoids

Casbene is widely recognized as the universal precursor from which macrocyclic diterpenoids such as ingenol are biosynthetically derived (**Figure 5**). First identified in *Ricinus communis*, casbene is produced from geranylgeranyl diphosphate (GGPP) by casbene synthase (CBS) (Hu et al., 2021). CBS genes have been functionally characterized from multiple Euphorbiaceae species, including *Euphorbia lathyris* (ElCBS), *E. esula* (EeTPS2), *Hyptis nutans* (HnTPS4), *E. resinifera* (ErTPS6), *Sapium sebiferum* (SsTPS10) (Luo et al., 2016; Hu et al., 2021). The consistent presence of casbene synthase activity across these species, coupled with the absence of alternative pathway entries, supports the role of casbene as the universal precursor for macrocyclic diterpenoids in this family. While casbene represents the primary gateway to ingenol-related pathways, a distinct macrocyclic scaffold is generated in *R. communis*, where neocembrene synthase (RcCAS2) converts GGPP to neocembrene (King et al., 2014). This olefin is subsequently oxidized by CYP726A15 to produce 5-keto-neocembrene, thereby expanding the repertoire of macrocyclic backbones available for downstream functionalization (King et al., 2014).

Further insights into the oxidative modifications that tailor the casbene scaffold have emerged from studies on *E. lathyris*. Transcriptomic analysis of mature seeds identified two CYP450s (CYP71D445 and CYP726A27) along with the ElADH1 (Luo et al., 2016). Together, these enzymes convert casbene to jolkinol C, a putative precursor of ingenol. Comparative transcriptomics in *E. peplus* revealed homologous enzymes (EpCBS, CYP71D365, CYP726A4, and EpADH1) with analogous functions, suggesting that the core biosynthetic machinery for macrocyclic diterpenoids is conserved across Euphorbia species.

Although the complete biosynthetic route to ingenol remains unresolved, downstream modifications have been partially characterized. In *E. lathyris*, two macrocyclic diterpenoid O-acyltransferases, ElBAHD16 and ElBAHD35, were identified from the diterpenoid biosynthetic gene cluster, and they exhibit mono-acylation activities toward the hydroxy groups of their substrates, 7-hydroxylathyrol and lathyrol (Zhao et al., 2025a). In *E. peplus*, heterologous expression and *in vitro* biochemical assays demonstrated that the products of EpBAHD-06 and EpBAHD-08 catalyze the addition of angelyl-CoA to the ingenol scaffold, yielding ingenol-3-angelate (Schotte et al., 2025). Virus-induced gene silencing (VIGS) experiments further revealed that EpBAHD-08 is essential for this angeloylation, whereas silencing of EpBAHD-06 predominantly affected jatrophane accumulation rather than ingenanes.

## 5 Key genes and TFs involved in diterpenoid biosynthesis

### 5.1 Phylogenetic analysis of diTPSs

**Figure 6:**
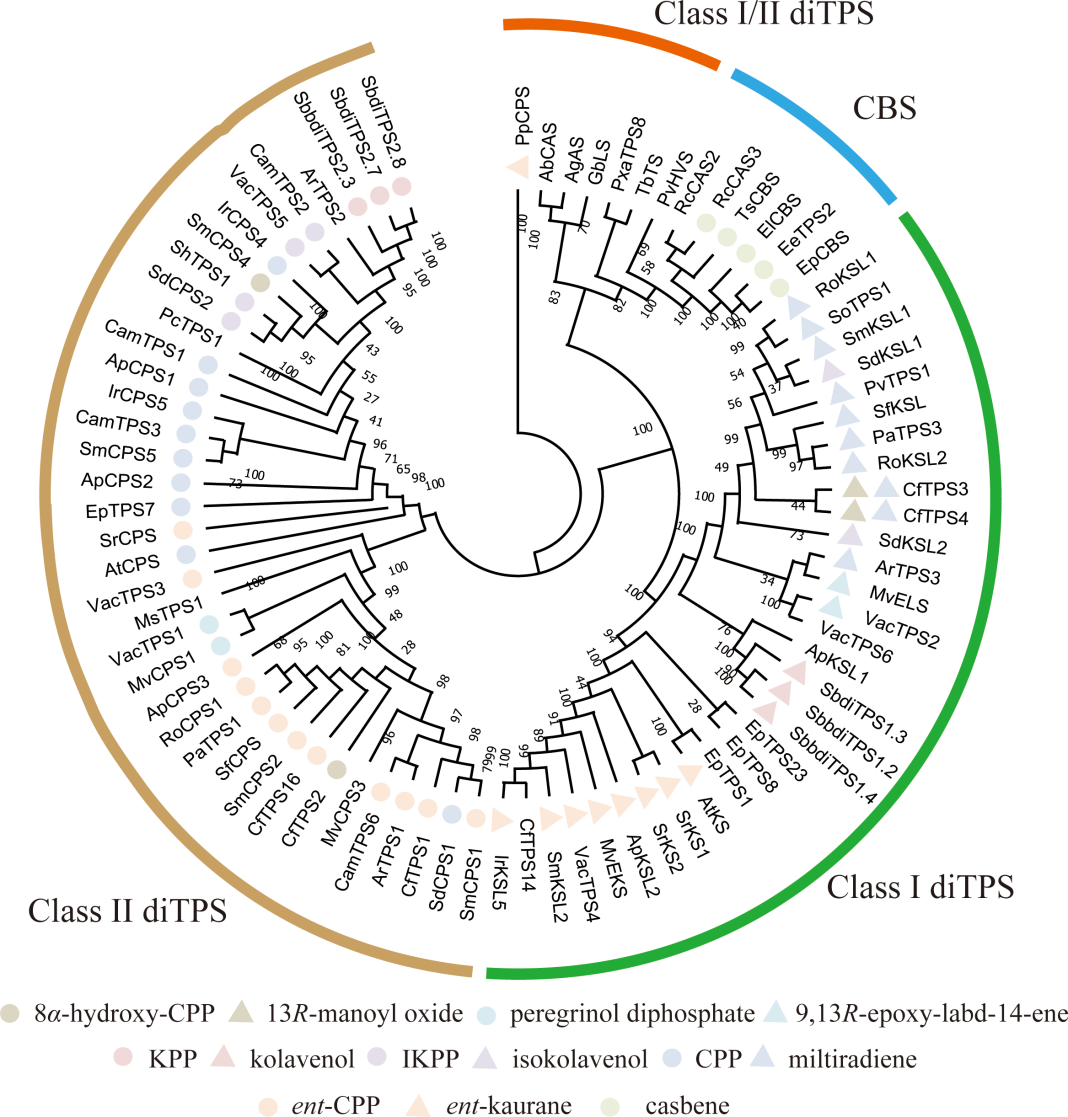
Phylogenetic tree of diTPSs involved in diterpenoid biosynthesis.

diTPSs serve as the first rate-limiting enzymes in diterpenoid biosynthesis, catalyzing the cyclization of GGPP into diverse hydrocarbon backbones. Structurally, most plant diTPSs adopt a three-domain (γ, β, α) architecture, although variations such as αβ or standalone α-domain variants also exist. Phylogenetic analysis using the neighbor-joining (NJ) method classified these enzymes into four distinct groups: Class I, Class II, bifunctional Class I/II, and CBS (**Figure 6**). Class II diTPSs are widely distributed across seed plants and directly convert GGPP into bicyclic scaffolds. They contain a DXDD motif at the γ-β interface for protonating the C-15 position (Liu et al., 2023). To date, eleven bicyclic backbones have been reported from these enzymes, including 8*β*-hydroxy-*ent*-CPP, peregrinol diphosphate, KPP, IKPP, CPP, and *ent*-CPP. Subsequently, these bicyclic scaffolds undergo diphosphate group removal and further cyclization catalyzed by Class I diTPSs. Class I diTPSs are reported in fewer plant lineages than Class II enzymes, being documented mainly in Lamiaceae, Euphorbiaceae, Acanthaceae, and Asteraceae. Structural studies have shown that Class I diTPSs utilize a DDXXD motif in the C-terminal α-domain to initiate metal-dependent ionization of the diphosphate group (Liu et al., 2023). This process generates tricyclic and tetracyclic diterpenoid skeletons, including labdane-type, clerodane-type, and other derivatives such as 13*R*-manoyl oxide, 9,13*R*-epoxy-labd-14-ene, kolavenol, isokolavenol, miltiradiene, and *ent*-kaurane. Notably, some Class I diTPSs exhibit multifunctional properties. For instance, CfTPS3 and CfTPS4 can utilize both 8*β*-hydroxy-ent-CPP and CPP as substrates, converting the former to 13*R*-manoyl oxide and the latter to miltiradiene (Pateraki et al., 2014). Unlike the functionally differentiated Class I and II enzymes in angiosperms, diTPSs in primitive gymnosperms remain undifferentiated, existing as bifunctional Class I/II variants that perform both cyclization and rearrangement in a single enzyme, thereby facilitating efficient substrate channeling. CBS has so far been identified exclusively in Euphorbiaceae plants and is involved in the biosynthesis of macrocyclic diterpenes such as casbene. Phylogenetic analysis reveals that Class II diTPSs are more distantly related to both bifunctional I/II and CBS, suggesting that Class II may have emerged later as functionally specialized enzymes with higher catalytic efficiency. Furthermore, reported species often contain multiple copies of Class I and Class II diTPSs, which is consistent with the structural diversity of diterpenoids.

### 5.2 Phylogenetic analysis of CYP450s

**Figure 7:**
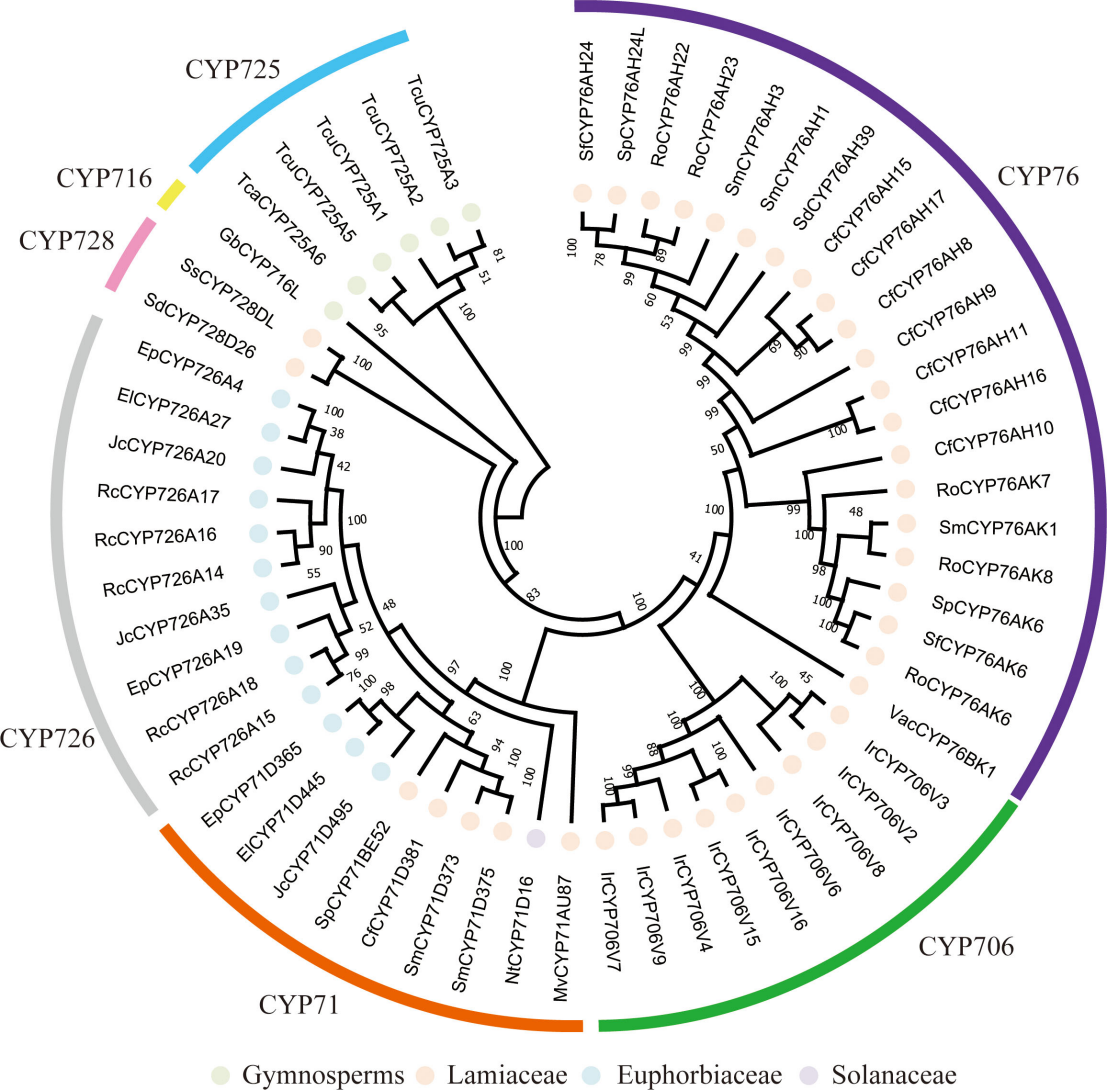
Phylogenetic tree of CYP450s involved in diterpenoid biosynthesis.

CYP450s are the primary enzymes responsible for the oxidative decoration of diterpene skeletons. This process dramatically expands the structural and functional diversity of this compound class (Frey et al., 2025). Phylogenetic analysis using the NJ method has revealed that reported CYP450 genes specifically involved in diterpenoid biosynthesis are mainly distributed across several families, including CYP71, CYP76, CYP706, CYP725, CYP726, and CYP728 (**Figure 7**). Among these CYP450s involved in diterpenoid biosynthesis, most are primarily involved in hydroxylation at various positions of diterpenoid skeletons, such as C-6, C-9, C-11, C-12, C-18, C-19, and C-20. Beyond these, certain CYP450s possess specialized activities. For example, SdCYP76AH39 and SmCYP71D375 are capable of forming furan rings; CfCYP76AH5, CfCYP76AH8, CfCYP76AH17, and RcCYP726A15 catalyze oxidation of the parent ring; and SdCYP728D26 and RoCYP76AK6–8 respectively facilitate C-20 carboxylation. Furthermore, CYP450s are typical paralogous gene families arising from gene duplication events within genomes (Bathe and Tissier, 2019). Phylogenetic analysis supports that their involvement in diterpenoid biosynthetic pathways correlates more strongly with plant lineages, with most reports concentrated in conifers, Lamiaceae, and Euphorbiaceae. Consequently, functional characterization of these CYP450s typically relies on comparative transcriptomics and gene cluster analysis, as they are often co-expressed with diTPSs and occasionally physically clustered in plant genomes, thus facilitating the discovery of complete biosynthetic pathways. Moreover, given their substrate promiscuity, CYP450s hold promise as potential biocatalysts for constructing non-native, highly water-soluble compounds.

### 5.3 Function of TFs

TFs play pivotal roles in the regulation of diterpenoid biosynthesis by modulating the expression of key biosynthetic genes. To date, several TF families have been implicated in this process across different plant species, including ERFs, bHLH, WRKY, NAC, MYC, and the less characterized WHIRLY family (Ishikawa et al., 2024; Lu et al., 2024; Zhou et al., 2024; Lin et al., 2025b). For instance, in *S. miltiorrhiza*, SmERF6 enhanced production of carnosol and carnosic acid by upregulating copalyl diphosphate synthase and KSL enzymes (Revuru et al., 2025), while SmERF105 promoted tanshinone biosynthesis by activating *KSL1*, *CYP76AH3*, and transporter *ABCG1* (Li et al., 2026). In contrast, the MYC2 TFs TwMYC2a and TwMYC2b from *Tripterygium wilfordii* function as negative regulators by repressing the transcription of miltiradiene synthase genes TwTPS27a and TwTPS27b, thereby suppressing triptolide biosynthesis (Huo et al., 2021). In tobacco, the WHIRLY family TF NtWHY1 directly binds the *NtCBTS* promoter to activate its expression, and positively correlates with both NtCBTS transcript levels and the accumulation of *α*- and *β*-cembrenediol, establishing NtWHY1 as a positive regulator of cembranoid diterpenoid biosynthesis (Zhai et al., 2025).

Rice presents a more complex regulatory landscape, wherein multiple TFs coordinately govern diterpenoid phytoalexin biosynthesis. OsbHLH5 functions as a positive bifunctional regulator that simultaneously activates the biosynthesis of both phenolamides and diterpenoid phytoalexins (Zhou et al., 2024). Overexpression of OsbHLH5 significantly elevates momilactones A and B levels, whereas knockout lines exhibit reduced accumulation of these compounds. Mechanistically, OsbHLH5 directly activates diterpenoid biosynthetic genes, including *OsCPS4* and multiple *OsKSL* family members. Other TFs in rice, such as OsWRKY10, OsNAC29, and OsDPF, have also been reported to contribute to diterpenoid phytoalexin accumulation through activation of biosynthetic genes (Zhou et al., 2024). Despite these advances, studies on transcriptional regulation of diterpenoid biosynthesis remain limited, likely due to incomplete pathway elucidation in many species. It is anticipated that ongoing efforts to characterize diterpenoid biosynthetic pathways will increasingly uncover the TFs that control them, ultimately providing valuable targets for metabolic engineering and crop improvement.

## 6 Heterologous biosynthesis increases accumulation of diterpenoids

**Figure 8:**
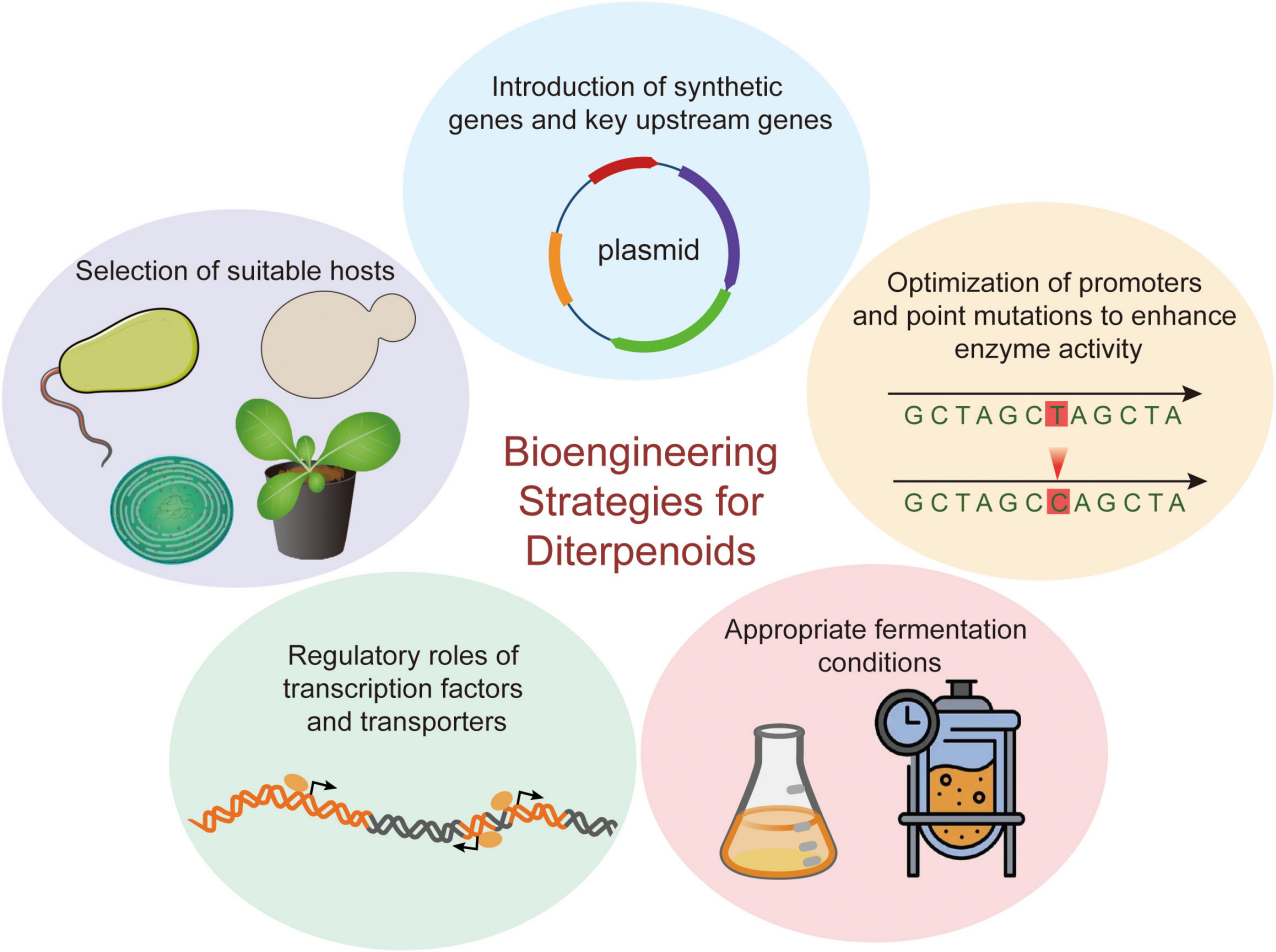
Bioengineering strategies for diterpenoids.

The structural complexity of diterpenoids often limits their commercial feasibility through direct extraction from native plant sources or total chemical synthesis. Heterologous biosynthesis has emerged as a promising alternative for the sustainable production of these high-value compounds. Over the past two decades, significant progress has been made in the reconstruction of plant-derived diterpenoid biosynthetic pathways within heterologous hosts. Key strategies to enhance diterpenoid biosynthesis include selecting suitable hosts, introducing synthetic and key upstream genes, optimizing promoters and introducing point mutations to boost enzyme activity, modulating TFs and transporters, and employing appropriate fermentation conditions (**Figure 8**).

### 6.1 Heterologous production in *E. coli*

*E. coli* offers several advantages for diterpenoid biosynthesis, including rapid growth, well-characterized genetics, high enzyme expression capacity, and abundant availability of precursor metabolite. Therefore, it serves as an effective platform for the functional characterization of diTPS enzymes. For example, in a Lamiaceae study, the transit peptides of diTPS proteins were removed based on ChloroP predictions, and the truncated versions were expressed in E. coli to assess their function (Li et al., 2023b). The biosynthesis of taxadiene, the precursor to paclitaxel, exemplifies successful hydrocarbon production in *E. coli*. A seminal study employing a multivariate-modular approach partitioned the pathway into upstream MEP modules and downstream taxadiene synthase modules (Ajikumar et al., 2010). Systematic balancing of these modules through combinatorial optimization of promoter strengths and gene copy numbers led to remarkable production improvements, achieving taxadiene production of 0.3 g/L in shake flask cultures and 1.0 g/L in fed-batch bioreactors (Ajikumar et al., 2010). Building on this foundation, subsequent work introduced a fusion protein combining a truncated taxadiene 5α-hydroxylase (CYP725A4) with its cognate cytochrome P450 reductase (CPR) from *Taxus* species, enabling production of 58 mg/L taxadien-5α-ol. While this strategy attempted to overcome the intrinsic challenge of P450 functional expression in prokaryotes, the yield of the oxygenated product remained substantially lower than that of the hydrocarbon precursor. In another example of successful hydrocarbon production, the complete biosynthetic pathway to sclareol was reconstructed in *E. coli* following its elucidation from *S. sclarea* (Schalk et al., 2012). This effort yielded 1.5 g/L sclareol in bioreactor cultivations, a production level approaching commercially relevant levels. Similarly, a production system for levopimaradiene, an intermediate of ginkgolides and industrial resin, was established in *E. coli* (Leonard et al., 2010). Combinatorial mutagenesis of both GGPP synthase and levopimaradiene synthase generated variant enzymes with enhanced catalytic properties, enabling production of 700 mg/L levopimaradiene in fed-batch fermentation.

However, as a prokaryotic system, it lacks the endomembrane infrastructure required for optimal eukaryotic P450 enzyme function—notably the absence of an endoplasmic reticulum and endogenous CPR partners (Liu et al., 2023). This limitation has largely restricted *E. coli* applications to the production of diterpene hydrocarbon skeletons rather than their fully oxygenated derivatives. Steviol glycoside production reached only 10.03 mg/L in engineered strains, a process that requires both P450-mediated oxidation and UGT activities (Wang et al., 2016b). This low production is attributed to the introduction of P450-containing plasmids, which consistently disrupt the carefully balanced metabolic networks optimized for hydrocarbon production.

### 6.2 Heterologous production in *S. cerevisiae*

Yeast has emerged as a preferred platform for producing complex oxygenated diterpenoids, owing to its eukaryotic endomembrane system, native CPR partners, and intrinsic mevalonate pathway for precursor supply. For example, the biosynthetic pathway of hautriwaic acid was elucidated through the co-expression of SsKPS, SsKLS, SsANS, SsHDAS, and HTAS in recombinant yeast (Lin et al., 2025a). In another study, the genes involved in cryptotanshinone biosynthesis were identified by expressing CYP71Ds in the WAT11 yeast strain (Ma et al., 2021). Significant engineering efforts have focused on taxadiene production in yeast. Initial attempts achieved 8.7 mg/L by overexpressing a truncated HMG-CoA reductase and introducing a heterologous GGPP synthase from *Sulfolobus* (Engels et al., 2008). Subsequent systematic optimization using a Cas9-based toolkit with combinatorial promoter and protein tag libraries increased production to 20 mg/L (Apel et al., 2017). When combined with low-temperature cultivation, yields were further elevated to 129 mg/L (Nowrouzi et al., 2020). Extension to oxygenated taxanes required the introduction of three *Taxus* genes: CYP725A4, CPR, and taxadien-5α-ol *O*-acetyltransferase. Optimization of oxygen and pH in controlled bioreactors enabled production of 78 mg/L total oxygenated taxanes, including 3.7 mg/L taxadien-5α-yl-acetate (Walls et al., 2021). An innovative microbial consortium strategy, establishing an engineered mutualistic relationship between *E. coli* and *S. cerevisiae*, yielded up to 30 mg/L oxygenated taxanes, including the advanced intermediate taxadien-5α-acetate-10β-ol (Zhou et al., 2015). Yeast engineering for steviol glycosides has also achieved notable success through sequential modular optimization. Kaurene-producing strains were initially developed by overexpressing kaurene synthase, truncated HMG-CoA reductase, isopentenyl diphosphate isomerase, and a mutant farnesyl diphosphate synthase (Xu et al., 2022a). Subsequent introduction of kaurenoic acid oxidase and kaurenoic acid 13-hydroxylase, along with their CPR partner, enabled steviol biosynthesis. The stepwise integration of UGTs culminated in the production of 1.369 g/L rubusoside and various rebaudiosides (Xu et al., 2022a).

This eukaryotic chassis has facilitated the successful reconstruction of numerous diterpenoid biosynthetic pathways that have not been reported in prokaryotic systems. For instance, sclareol production was enhanced through heterologous expression of GGPP synthase and mutation of endogenous farnesyl diphosphate synthase, yielding 403 mg/L in shake flasks (Ignea et al., 2015a, 2015). A carotenogenic screen subsequently identified beneficial gene deletions, and the resulting sextuple-gene mutant nearly doubled production to 750 mg/L in shake flasks (Trikka et al., 2015). Similarly, miltiradiene, a key intermediate in the biosynthesis of triptolide, tanshinones, and carnosic acid, has been produced in yeast through comprehensive modular engineering. The pathway was divided into three modules: diTPSs (SmKSL-SmCPS), prenyl transferases (BTS1-ERG20), and the tHMG1 regulatory module (Zhou et al., 2012). This modular strategy enabled rapid assembly and optimization, resulting in a miltiradiene yield of 365 mg/L. Subsequent refinements were then implemented, including flux redirection, transcriptional regulator manipulation, and protein fusion optimization. These synergistic approaches further boosted production to an unprecedented 3.5 g/L (Hu et al., 2020). In studies on carnosic acid, the initial yield reached only 1 mg/L upon insertion of a mutant farnesyl diphosphate synthase fused with copalyl diphosphate synthase in yeast (Ignea et al., 2016a). Successive improvements were implemented, including linker optimization, cofactor balancing, and detoxification of reactive byproducts. Consequently, carnosic acid production was increased to 24.7 mg/L in shake flasks and 75.2 mg/L in fed-batch bioreactors (Wei et al., 2022). For forskolin, mutation of the substrate recognition site 1 region of CYP76AH15 improved catalytic efficiency, leading to enhanced accumulation of pathway intermediates (Forman et al., 2018). Additionally, deletion of host genes such as *mct1*, *whi2*, and *gdh1* boosted production of the forskolin precursor 11-β-hydroxy-manoyl oxide by 9.5-fold (Ignea et al., 2016b). In addition, the macrocyclic diterpenoid jolkinol C was produced in yeast through the introduction of seven pathway genes, achieving 800 mg/L (Wong et al., 2018). Separately, feeding tanshinone IIA to yeast expressing codon-optimized *SmCYP71D373* at 28 °C for 24 hours yielded up to 6.38% tanshinone IIB (Qiu et al., 2024a).

Collectively, these successes underscore the remarkable versatility of yeast as a platform for complex diterpenoid biosynthesis. As more plant pathways are elucidated and synthetic biology toolkits continue to expand, yeast is poised to enable the scalable production of an ever-widening array of high-value diterpenoids for pharmaceutical and industrial applications.

### 6.3 Heterologous production in plant systems

Plant systems offer distinct advantages for diterpenoid production, including appropriate subcellular compartmentalization, native redox partners for CYP450s, and established protein processing machinery required for plant enzyme function. Metabolic engineering of *S. miltiorrhiza* hairy roots through co-expression of endogenous HMGR and GGPP synthase achieved a 4.74-fold increase in tanshinone accumulation (Ikram and Simonsen, 2017). Additionally, RNAi-mediated repression of 2-*ODD14* in hairy roots enhanced dihydrofuran-tanshinones but reduced furan-tanshinones (Song et al., 2022), while overexpression of SmCYP81C16 in hairy roots promoted (iso)tanshinone accumulation (Ren et al., 2023). In *E. lathyris*, ElBAHD16 overexpression increased 7-O-acetyllathyrol and 7-O-benzoyllathyrol, whereas ElBAHD35 elevated 5-O-acetyl-7-hydroxylathyrol and 5-O-acetyllathyrol in hairy roots (Zhao et al., 2025a). Separately, VIGS of *EpBAHD-08* in *E. peplus* verified its function in ingenol C3 angeloylation to yield ingenol-3-angelate (Schotte et al., 2025).

While this approach effectively leverages the native metabolic infrastructure within producing species, its broader application is constrained by variable susceptibility to *Agrobacterium*-mediated transformation among medicinal plants. Tobacco has consequently emerged as a versatile platform for evaluating enzyme function, enabling transient expression of pathway combinations. Chloroplast compartmentalization of taxadiene biosynthesis, combined with MEP pathway enhancement, yielded 56.6 μg/g taxadiene and 1.3 μg/g taxadiene-5α-ol (Li et al., 2019). Transient expression of diTPSs from *Scutellaria barbata*, *S. baicalensis*, and *Salvia splendens* in tobacco enabled functional assignment (Li et al., 2023b). Furthermore, the tobacco expression system revealed that IrCYP706V2 and IrCYP706V7 catalyze six regiospecific oxidations in oridonin biosynthesis (Sun et al., 2023). This system demonstrates the utility of plant-based platforms for rapid enzyme variant testing, despite achieving lower absolute yields compared to microbial systems.

Beyond traditional plant systems, photosynthetic microorganisms have emerged as promising alternatives for sustainable diterpenoid production. The green alga *Chlamydomonas reinhardtii* represents an innovative platform in this context. By identifying and engineering bottlenecks in the MEP pathway through enzyme fusion strategies, researchers significantly enhanced metabolic flux. When coupled with photoautotrophic high-cell-density fermentation, this approach yielded 656 mg/L sclareol after 19 days of cultivation (Einhaus et al., 2022). In another example, heterologous expression of *DgTPS1* in *Nannochloropsis oceanica*, combined with engineering of endogenous 1-deoxy-D-xylulose-5-phosphate synthase and geranylgeranyl diphosphate synthase genes, enabled casbene production reaching 1.80 mg/g total dry cell weight (Du et al., 2023). These achievements demonstrate the potential of microalgae to harness light and carbon dioxide for sustainable diterpenoid biosynthesis, offering a production route that avoids the land-use and seasonal constraints associated with terrestrial plant cultivation. Although still in early stages of development, such photosynthetic platforms complement microbial systems by providing environmentally sustainable production alternatives.

## 7 Discussion

### 7.1 Enhanced biological activities: mechanistic insights and structural modification

Diterpenoids exhibit significant anti-inflammatory, anticancer, and antimicrobial activities, with several members (e.g., andrographolide, tanshinone, paclitaxel, abietic acid) already approved as therapeutics. In recent years, an increasing number of newly reported diterpenoids have exhibited strong biological activities (**Table 1**). To realize this therapeutic potential, standardized testing protocols using consistent units and comparator strains are urgently needed to enable cross-study comparisons. For anti-infective candidates, evaluation should extend beyond *in vitro* potency to include efficacy, pharmacokinetics, and toxicity in appropriate animal infection models. In addition, a more comprehensive assessment of therapeutic potential requires investigating their effects on biofilms, mixed-species communities, synergy with existing antibiotics, and activity against intracellular pathogens.

Beyond discovering new bioactive diterpenoids, future efforts should prioritize improving the water solubility and target affinity of active compounds while reducing their cytotoxicity. However, such research remains limited for diterpenoids, constraining their clinical application. Abietic acid (203), traditionally used as a topical antibacterial agent, has also been shown to exhibit anticancer, anti-inflammatory, and hepatoprotective effects via ferroptosis, the COX-2/PPAR pathways, and the Nrf2/HO-1 pathway (Ito et al., 2020; Ren et al., 2026). Nevertheless, its clinical translation is limited by poor solubility, low oral bioavailability, and high first-pass metabolism. Chemical modification offers a viable strategy to address these issues. For instance, columbin (127) activates PXR similarly to rifampicin (Parveen et al., 2022) but induces dose-dependent hepatotoxicity in mice, an effect absent with tetrahydrocolumbin (Huang et al., 2025).

Beyond chemical modification, advanced formulation strategies such as liposomes and 3D printing can enhance drug efficacy. Liposomes are nanoscale phospholipid carriers that enhance efficacy and reduce side effects via biocompatibility and targeting, and are widely used in antitumor, anti-infective, and vaccine applications. 3D printing fabricates drugs layer-by-layer from digital models, enabling personalized dosing, complex geometries, and rapid production, with significant potential for rare diseases, pediatrics, and dysphagia. For example, long-circulating liposomes loaded with oridonin (294) and engineered to target lung cancer cells significantly inhibited tumor growth in nude mice bearing A549 cell xenografts (Han et al., 2025; Shi et al., 2026). Furthermore, biosynthetic approaches leveraging the hydroxylation capability of CYP450s can also improve the water solubility of bioactive compounds. Collectively, structural modification represents a promising strategy to reduce toxicity, improve bioavailability, and advance the development of novel diterpenoid-based drugs.

### 7.2 Pathway elucidation: omics analysis and large-scale screening

Diterpenoids have become hotspots for pathway elucidation due to their diverse structures and significant bioactivities. Omics technologies have recently accelerated pathway discovery through a streamlined workflow: RNA-Seq and genome sequencing generate high-precision data, bioinformatics algorithms rapidly identify candidate genes, and transient expression in *E. coli*, yeast, or tobacco enables functional validation of multi-gene combinations (Tian et al., 2025; Chen et al., 2026). Data mining is critical to this process, with common strategies including co-expression analysis with upstream diTPSs, mining genomic gene clusters, and integrating metabolomics to infer enzymatic reactions. These approaches have enabled partial or complete pathway characterization for several important diterpenoids, including forskolin, tanshinones, oridonin, steviol glycosides, and casbene-derived macrocyclic diterpenoids (**Figures 4**, **5**). Nevertheless, most pathways remain incomplete, particularly regarding downstream genes—a bottleneck that limits metabolic engineering for diterpenoid production.

Diterpenoids constitute a large class of natural products with over 12,000 known structures, of which more than 1,000 possess beneficial bioactivities and are widely distributed across medicinal plants. Therefore, pathway elucidation should not be confined to the Lamiaceae family; other families with unique diterpenoid skeletons, such as Euphorbiaceae, Ericaceae, and Fabaceae, also warrant in-depth investigation. Investigating multiple compounds can offer new insights for pathway discovery. The distribution and pharmacological activity summarized in this review may serve as a reference for selecting study targets (**Table 1**). Beyond mining enzymes with high sequence similarity, attention should be given to those distantly related to known sequences—particularly those sharing less than 30% identity with characterized terpene synthases—as they may possess entirely novel functions. Moreover, even enzymes with high sequence similarity can exhibit divergent activities. Therefore, systematic bioinformatics analysis must be complemented by large-scale experimental screening.

### 7.3 Diterpenoid overproduction: modular biosynthesis and multifaceted engineering

Several major constraints hinder the efficient overproduction of diterpenoids. First, as secondary metabolites, the imbalance in metabolic flux between precursor supply and downstream consumption restricts yields. Second, the vast structural diversity of diterpenoids means that most biosynthetic pathways remain incompletely elucidated. Third, the promiscuity of biosynthetic enzymes often leads to byproduct formation, reducing the yield of target molecules. Addressing these challenges requires an integrated approach combining modular biosynthesis with multiple engineering strategies, including genetic and enzyme engineering, cell engineering, and fermentation engineering.

Diterpenoids, owing to their structural complexity, are particularly well-suited for modular biosynthesis. A typical design divides the pathway into three modules: scaffold construction, oxidative modification, and post-modification. Cross-combination of diTPS and CYP450 modules from different species enables rapid generation of diverse diterpenoid libraries. By extension, drawing inspiration from the semi-synthesis of paclitaxel (Brunáková et al., 2026), integrating plant, microbial, and chemical steps offers a practical route for bioactive diterpenoids whose complete biosynthesis remains challenging.

At the genetic and enzyme engineering levels, optimization strategies include promoter modification, gene copy number regulation, and codon optimization. Furthermore, site-directed mutagenesis of key genes represents a powerful approach to optimize product profiles and enhance target diterpene yields (Hu et al., 2021; Li et al., 2023a). Specifically, guided by protein structural information of homologous enzymes from different species, point mutations in diTPSs and CYP450s offer further opportunities to improve the production of target diterpenoids. At the cellular engineering level, optimization strategies include modifying and regulating endogenous pathways to increase precursor supply, suppressing competing pathways via CRISPR/Cas9-mediated genome editing, streamlining the host genome, modulating transporter proteins to alleviate metabolite transport limitations, and regulating TFs. Meanwhile, emerging strategies such as endoplasmic reticulum engineering and subcellular organelle construction offer new avenues to improve chassis cell performance and reduce product toxicity. Furthermore, at the fermentation engineering level, systematic control of fermentation parameters, including pH, dissolved oxygen, induction timing, and carbon/nitrogen feeding strategies, enhances production efficiency. Co-culture fermentation combined with modular biosynthesis is also an important strategy. Notably, strategic combination of enzymes from different species enables the production of novel metabolites not found in nature, promising a future of “directed biosynthesis” akin to chemical synthesis. Finally, advances in robotics have enabled automated biofoundries that perform end-to-end automation of gene cloning, strain construction, and fermentation screening, dramatically increasing experimental throughput.

### 7.4 Efficient platforms: machine learning for pharmacology, biosynthesis, and enzyme engineering

Artificial intelligence, particularly machine learning, is transforming natural product research across pharmacology, biosynthetic pathway discovery, and enzyme engineering. In the field of natural product pharmacology, several databases—including TCMSP, NPBS Atlas, GNDC, BATMAN-TCM 2.0, and DCABM-TCM—have been developed to analyze ADME parameters, screen drug-like components, explore natural product distribution, discover novel scaffolds, construct network pharmacological maps, predict targets, and assess potential toxicity risks. However, these platforms face significant limitations, including insufficient authority, lack of continuous updates, and variable reliability, with different databases often yielding inconsistent predictions and covering only a fraction of disease associations validated by *in vivo* studies. Machine learning is poised to address these challenges by enabling the integration of multi-source data and improving prediction accuracy. Recently, machine learning tools—including tree ensembles, graph neural networks, and self-supervised molecular embeddings—have been applied to predict anticancer, anti-inflammatory, and antimicrobial activities. Looking forward, integrating machine learning with experimental models holds the potential to transform pharmacology from a descriptive science into a predictive one.

Beyond pharmacology, machine learning is revolutionizing biosynthetic pathway discovery and design. Machine learning models can extract hidden pathways from massive genomic and metabolomic datasets, predict unknown enzyme functions and catalytic mechanisms, and even design entirely novel enzymes not found in nature. A notable example is RxnNet, an AI-assisted platform for automated reaction mechanism discovery that generates complete reaction pathways and free energy landscapes from only the SMILES structures of substrates and products. Such tools hold immense promise for accelerating the elucidation of complex diterpenoid biosynthetic pathways, particularly for those involving cryptic steps or reactive intermediates.

AlphaFold and related machine learning technologies are enhancing the research efficiency of enzyme engineering to improve catalytic efficiency, stability, and substrate specificity. In the study of structural diversification of *Aconitum* diterpenoids, AlphaFold2 prediction of functionally divergent CYP450 enzyme structures enabled comparative structural analysis and site-directed mutagenesis, leading to the identification of key amino acid residues affecting functional differentiation and catalytic activity (Luo et al., 2025). This case demonstrates that machine learning-driven protein engineering can efficiently optimize enzyme performance even for enzymes lacking solved crystal structures or sharing only distant sequence homology with characterized proteins. Furthermore, the integration of machine learning with automated experimental platforms will further accelerate the cycle of structure prediction, mutation design, functional validation, and iterative optimization. This will make enzyme engineering faster, cheaper, and more predictable, thereby accelerating the generation of high-performance engineered strains.

In summary, the integration of synthetic biology, artificial intelligence, and metabolic engineering will enable scalable production of an increasingly diverse range of high-value diterpenoids for pharmaceutical and industrial applications. Sustained efforts in pathway elucidation, enzyme characterization, and chassis development will progressively overcome current limitations. With heightened global awareness of antimicrobial resistance and the urgent need for sustainable therapeutic production, medicinal plant diterpenoids are poised to play an increasingly vital role in addressing healthcare challenges and emerging biomedical needs.

## Supplementary material

Supplementary Figure 1 Structures of diterpenoid compounds in **Table 1**.

Supplementary Table 1 Genetic information in the phylogenetic tree of diTPSs.

Supplementary Table 2 Genetic information in the phylogenetic tree of CYP450s.
