# Supplementary material for: Diterpenoids in medicinal plants: structure, distribution, biological activities, biosynthesis, and bioengineering prospects
Source: Front Plant Sci. 2026 May 19;17:1833614. doi: 10.3389/fpls.2026.1833614 (PMC13226630; doi:10.3389/fpls.2026.1833614)
Supplement: Supplementary Figure 1 — Structures of diterpenoid compounds in Table 1. [file SupplementaryFile1.docx]

Supplementary Material

**Supplementary Figure 1.** Structures of diterpenoid compounds in table 1


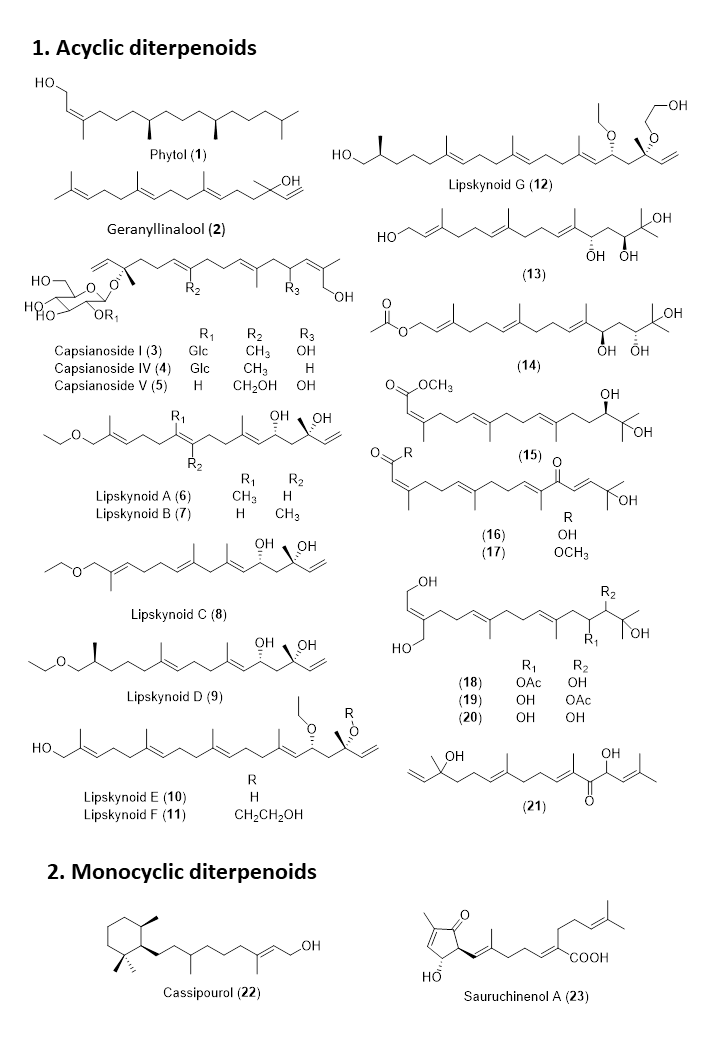


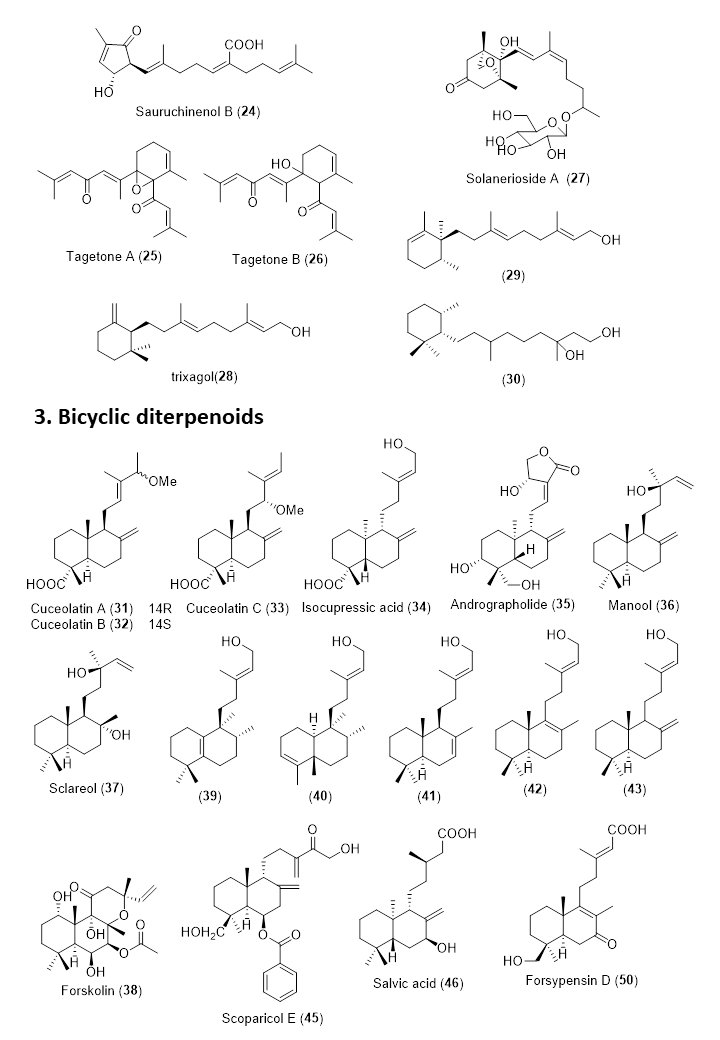

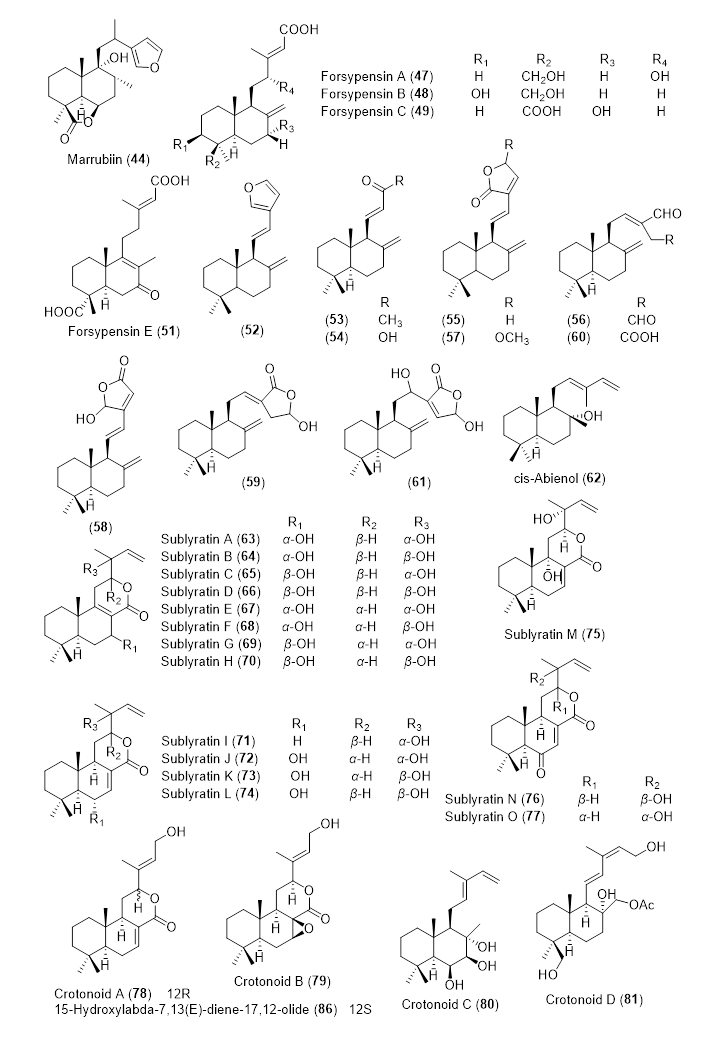

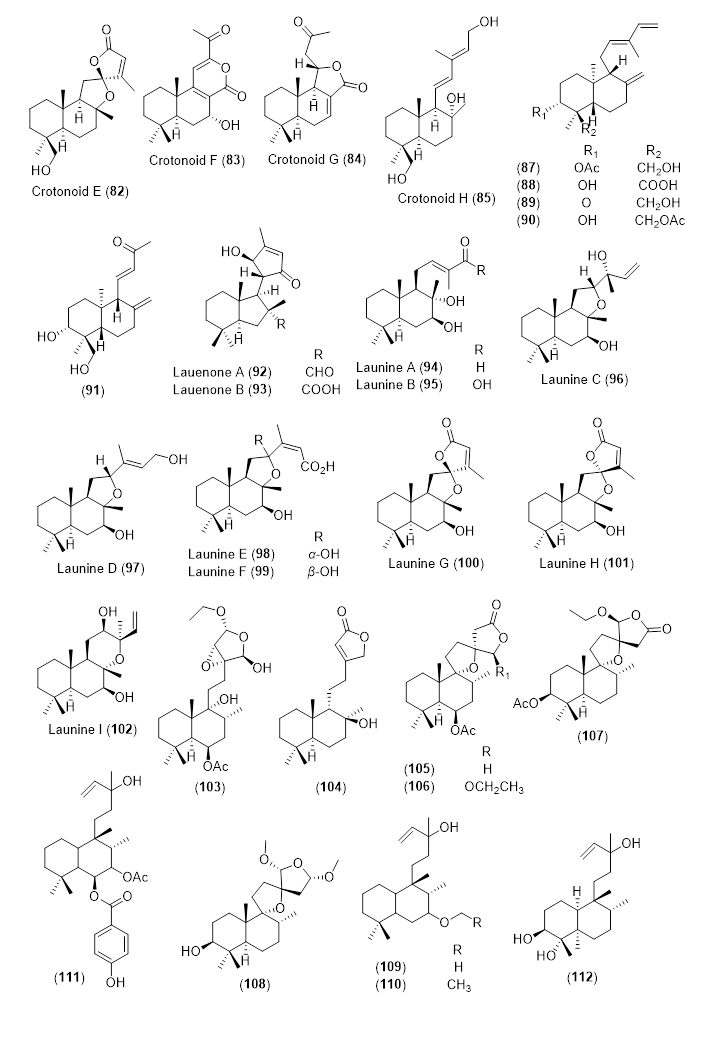

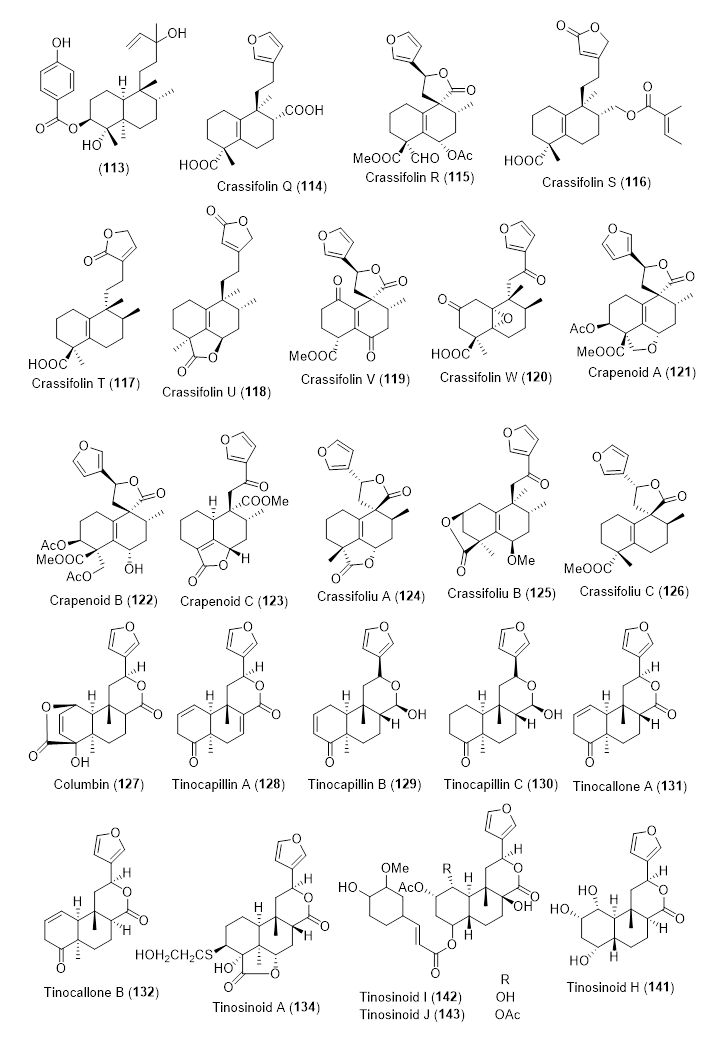

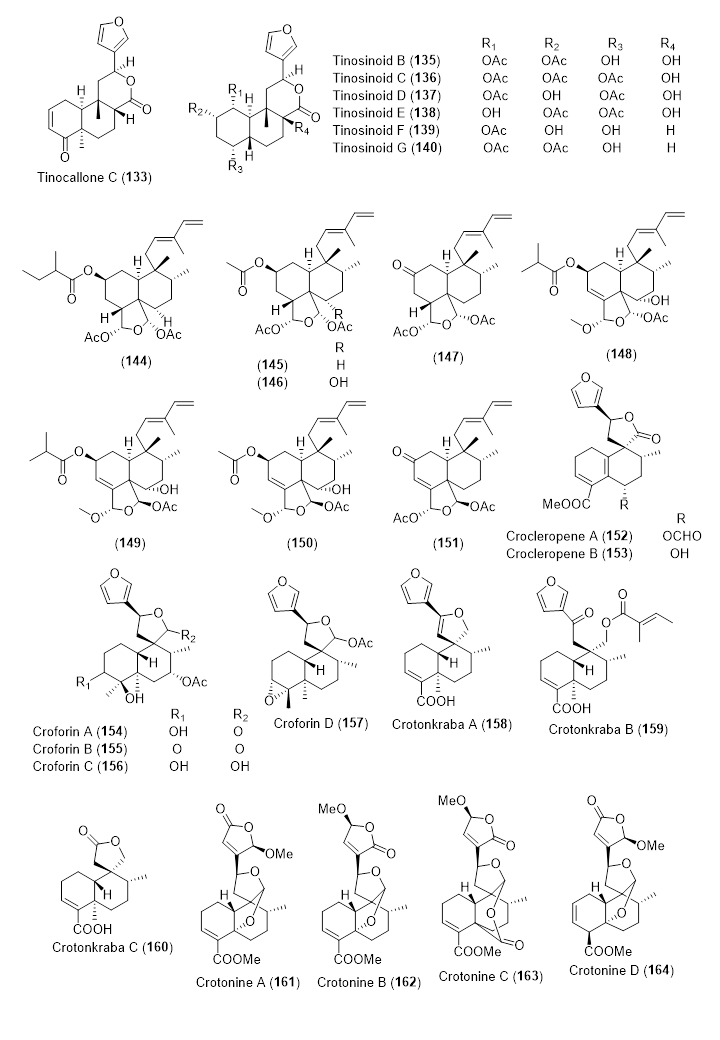

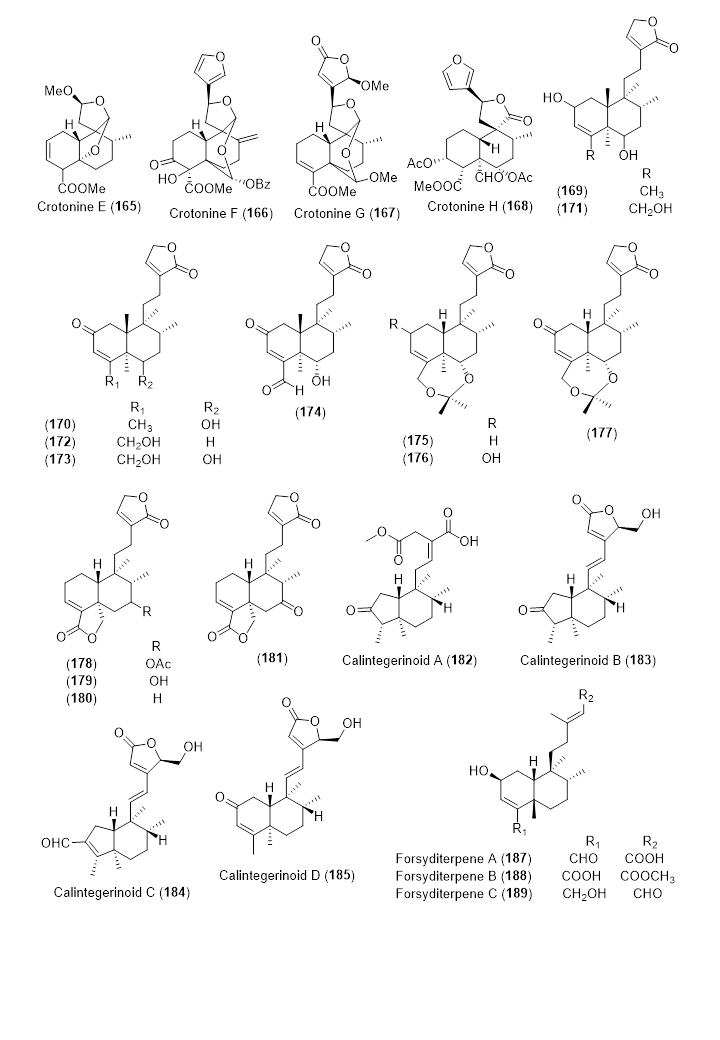

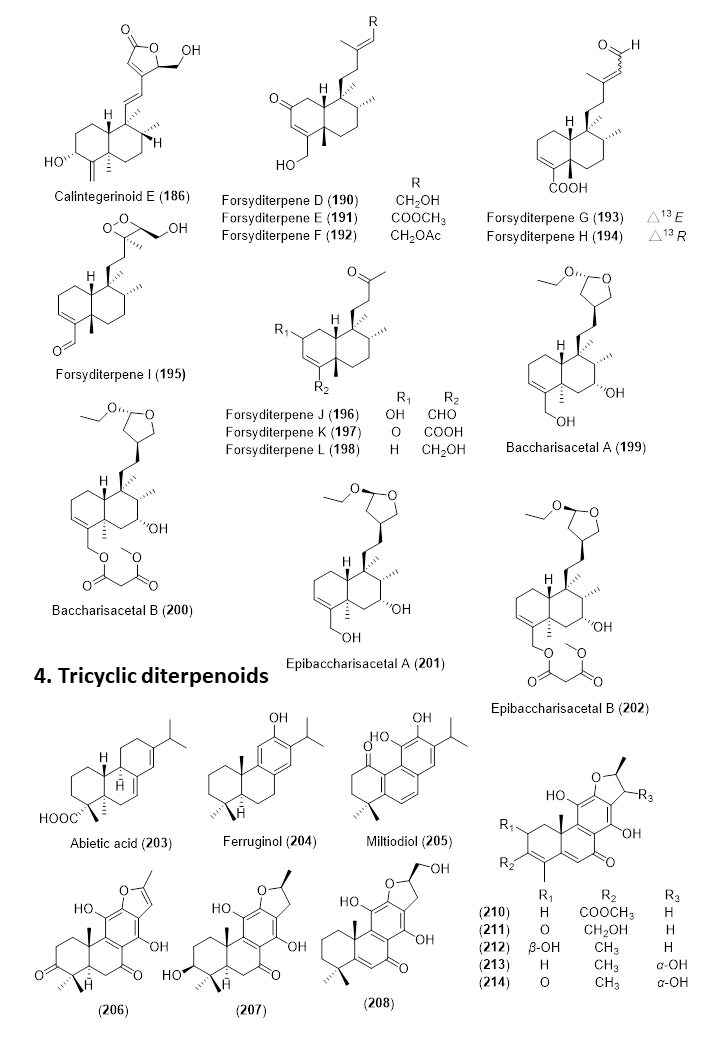


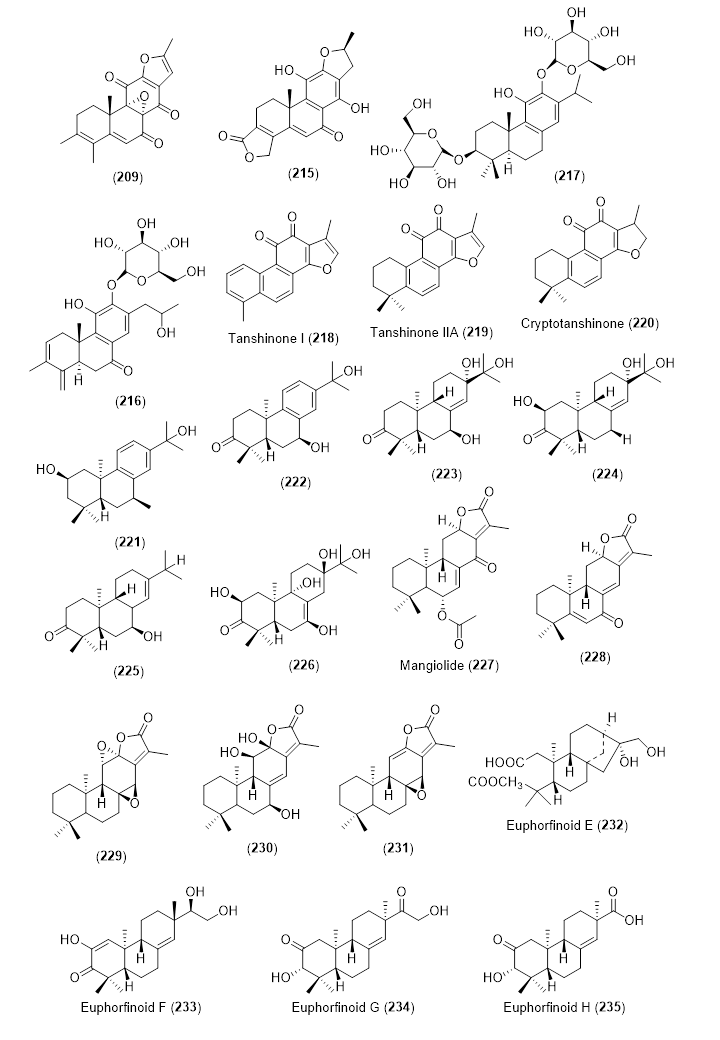


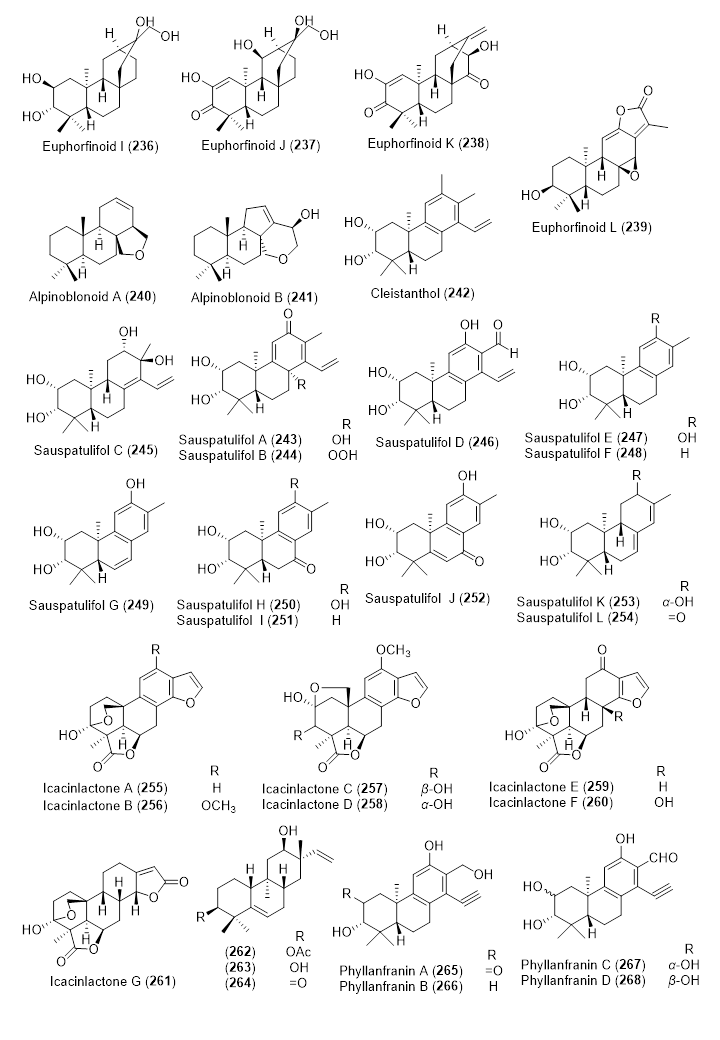


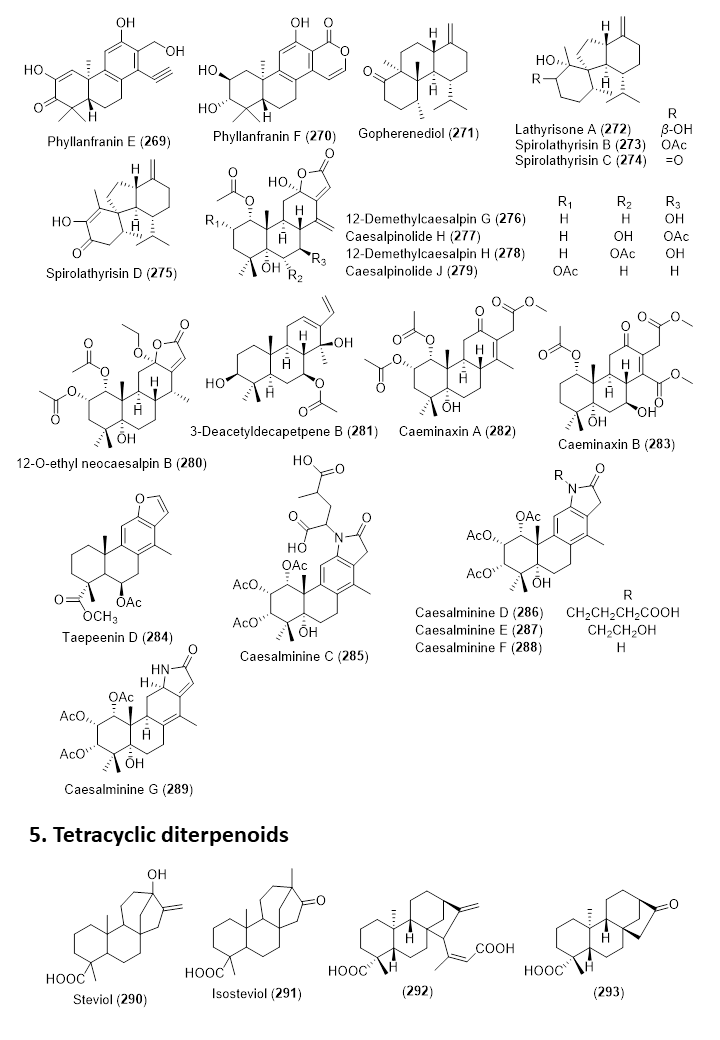


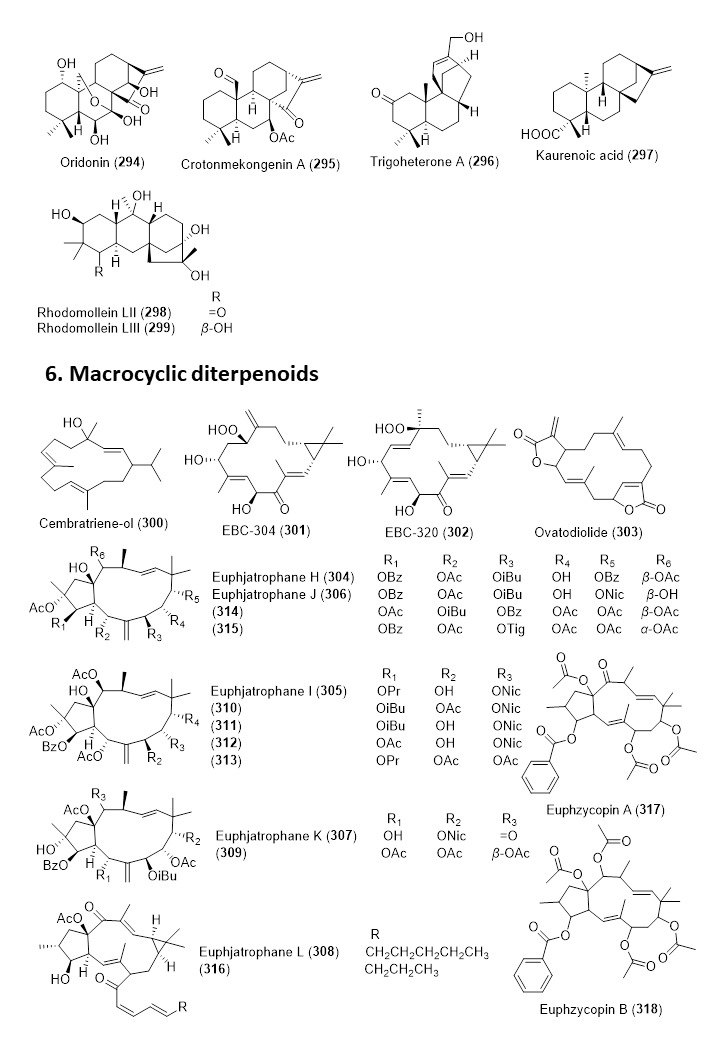


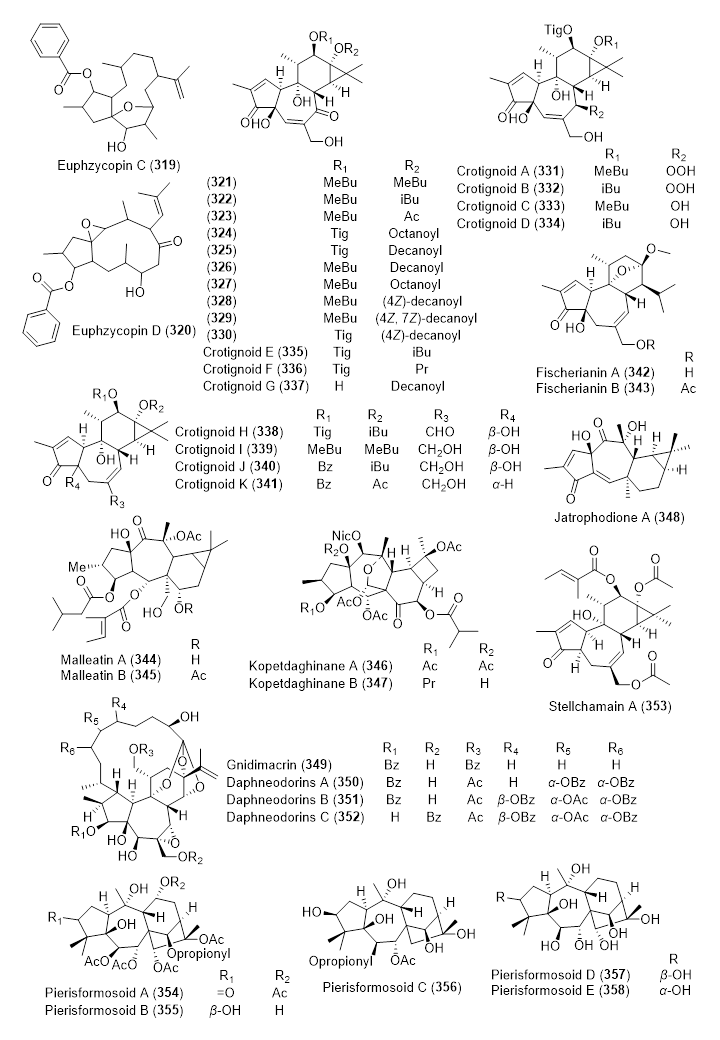

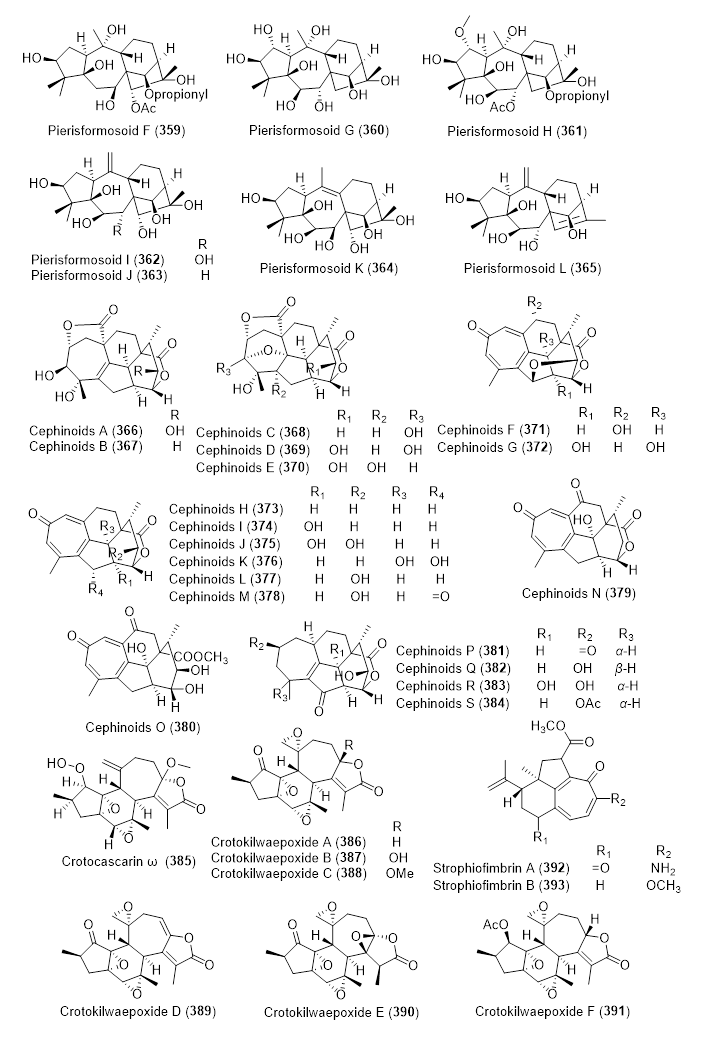


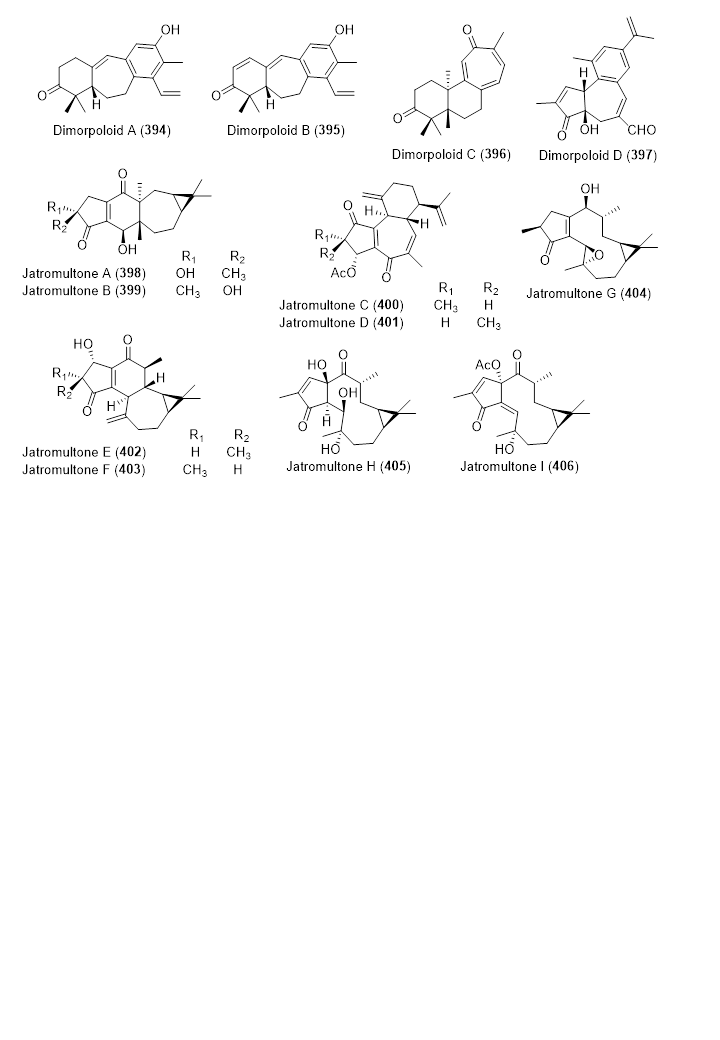


**Supplementary Table 1.** Genetic information in the phylogenetic tree of diTPSs

| Gene | Species | GenBank Accession | Products |
| --- | --- | --- | --- |
| AbCAS | *Abies balsamea* | AEL99953 | *cis*-abienol |
| AgAS | *Abies grandis* | AAB05407 | abietadiene |
| ApCPS1 | *Andrographis paniculata* | AFH53507 | *ent*-CPP |
| ApCPS2 | *A. paniculata* | AFH53508 | *ent*-CPP |
| ApCPS3 | *A. paniculata* | AXL95252 | CPP |
| ApKSL1 | *A. paniculata* | AXL95253 | isopimara-7,15-diene |
| ApKSL2 | *A. paniculata* | AXL95254 | *ent*-kaurane |
| ArTPS1 | *Ajuga reptans* | AZB50377 | CPP |
| ArTPS2 | *A. reptans* | AZB50378 | *neo*-cleroda-4(18),13*E*-dienyldiphosphate |
| ArTPS3 | *A. reptans* | AZB50367 | miltiradiene |
| AtCPS | *Arabidopsis thaliana* | Q38802 | *ent*-CPP |
| AtKS | *A. thaliana* | Q9SAK2 | *ent*-kaurane |
| CamTPS1 | *Callicarpa americana* | QMW69081 | *ent*-CPP |
| CamTPS2 | *C. americana* | QMW69082 | KPP |
| CamTPS3 | *C. americana* | QMW69080 | *ent*-CPP |
| CamTPS6 | *C. americana* | QMW69083 | CPP |
| CfTPS1 | *Coleus forskohlii* | KF444506 | CPP |
| CfTPS14 | *C. forskohlii* | AGN70881 | *ent*-kaur-16-ene |
| CfTPS16 | *C. forskohlii* | AZB50379 | CPP |
| CfTPS2 | *C. forskohlii* | AHW04047 | 8*α*-hydroxy-CPP |
| CfTPS3 | *C. forskohlii* | AHW04048 | 13*R*-manoyl oxide; miltiradiene |
| CfTPS4 | *C. forskohlii* | AHW04049 | 13R-manoyl oxide; miltiradiene |
| EeTPS2 | *Euphorbia Esula* | ADB90273 | casbene |
| ElCBS | *Euphorbia lathyris* | AMY98417 | casbene |
| EpCBS | *Euphorbia peplus* | AGN70884 | casbene |
| EpTPS1 | *E. peplus* | AGN70882 | *ent*-kaur-16-ene |
| EpTPS23 | *E. peplus* | ALE19953 | *ent*- sandaracopimaradiene |
| EpTPS7 | *E. peplus* | AGN70883 | *ent*-CPP |
| EpTPS8 | *E. peplus* | ALE19952 | sandaracopimaradiene |
| GbLS | *Ginkgo biloba* | AAL09965 | levopimaradiene |
| IrCPS4 | *Isodon rubescens* | APJ36374 | *ent*-CPP |
| IrCPS5 | *I. rubescens* | APJ36375 | *ent*-CPP |
| IrKSL5 | *I. rubescens* | ASC55317 | *ent*-kaurane |
| MsTPS1 | *Mentha spicate* | AZB50369 | sandaracopimaradiene |
| MvCPS1 | *Marrubium vulgare* | AIE77090 | peregrinol diphosphate |
| MvCPS3 | *M. vulgare* | AIE77092 | CPP |
| MvEKS | *M. vulgare* | AIE77093 | *ent*-kaurane |
| MvELS | *M. vulgare* | AIE77094 | 9,13*R*-epoxy-labd-14-ene |
| PaTPS1 | *Perovskia. atriplicifolia* | AZB50384 | CPP |
| PaTPS3 | *P. atriplicifolia* | AZB50374 | miltiradiene |
| PcTPS1 | *Pogostemon cablin* | AZB50385 | 10*R*-labda-8,13*E*-dienyl diphosphate |
| PpCPS/KS | *Physcometrium patens* | BAF61135 | *ent*-kaurane |
| PvHVS | *Prunella vulgaris* | AZB50511 | 11-hydroxy vulgarisane |
| PvTPS1 | *P. vulgaris* | AZB50375 | miltiradiene |
| PxaTPS8 | *Pseudolarix amabilis* | APT40486 | pseudolaratriene |
| RcCAS2 | *Rosmarinus communis* | XP_002513334.4 | neocembrene |
| RcCAS3 | *R. communis* | XP_002513343.2 | casbene |
| RoCPS1 | *Rosmarinus officinalis* | AHL67261 | CPP |
| RoKSL1 | *R. officinalis* | AHL67262 | miltiradiene |
| RoKSL2 | *R. officinalis* | AHL67263 | miltiradiene |
| SbbdiTPS1.2 | *Scutellaria barbata* | UNZ11783 | isokolavenol |
| SbbdiTPS1.4 | *S. barbata* | UNZ11785 | isokolavenol |
| SbbdiTPS2.3 | *S. barbata* | UNZ11788 | IKPP |
| SbdiTPS1.3 | *Scutellaria baicalensis* | UNZ93471 | isokolavenol |
| SbdiTPS2.7 | *S. baicalensis* | UNZ93478 | IKPP |
| SbdiTPS2.8 | *S. baicalensis* | UNZ93479 | IKPP |
| SdCPS1 | *Salvia divinorum* | APH81399 | *ent*-CPP |
| SdCPS2 | *S. divinorum* | APH81400 | KPP |
| SdKSL1 | *S. divinorum* | APG42602 | kolavenol |
| SdKSL2 | *S. divinorum* | APG42603 | kolavenol |
| SfCPS | *Salvia fruticosa* | AJQ30184 | CPP |
| SfKSL | *S. fruticosa* | AJQ30185 | miltiradiene |
| ShTPS1 | *Salvia hispanica* | XP_047942076 | KPP |
| SmCPS1 | *Salvia miltiorrhiza* | AHJ59321 | CPP |
| SmCPS2 | *S. miltiorrhiza* | AHJ59322 | CPP |
| SmCPS4 | *S. miltiorrhiza* | AKN91186 | 8*α*-hydroxy-CPP |
| SmCPS5 | *S. miltiorrhiza* | AHJ59324 | *ent*-CPP |
| SmKSL1 | *S. miltiorrhiza* | ABV08817 | miltiradiene |
| SmKSL2 | *S. miltiorrhiza* | AHJ59325 | *ent*-kaurane |
| SoTPS1 | *Salvia officinalis* | AZB50376 | miltiradiene |
| SrCPS | *Stevia rebaudiana* | AAB87091 | *ent*-CPP |
| SrKS1 | *S. rebaudiana* | AAD34294 | *ent*-kaurane |
| SrKS2 | *S. rebaudiana* | AAD34295 | *ent*-kaurane |
| TbTS | *Taxus brevifolia* | AAC49310 | taxadiene |
| TsCBS | *Triadica sebifera* | ADB90272 | casbene |
| VacTPS1 | *Vitex agnus-castus* | AUT77120 | peregrinol diphosphate |
| VacTPS2 | *V. agnus-castus* | AUT77121 | 9,13*R*-epoxy-labd-14-ene |
| VacTPS3 | *V. agnus-castus* | AUT77122 | CPP |
| VacTPS4 | *V. agnus-castus* | AUT77123 | *ent*-kaurane |
| VacTPS5 | *V. agnus-castus* | AUT77124 | KPP |
| VacTPS6 | *V. agnus-castus* | AUT77125 | labd-13(16),14-diene-9-ol |

**Supplementary Table 2.** Genetic information in the phylogenetic tree of CYP450s

| Gene | Species | GenBank Accession |
| --- | --- | --- |
| CfCYP71D381 | *Coleus forskohlii* | AMZ03386.1 |
| CfCYP76AH10 | *C. forskohlii* | AMZ03390.1 |
| CfCYP76AH11 | *C. forskohlii* | AMZ03393.1 |
| CfCYP76AH15 | *C. forskohlii* | AMZ03402.1 |
| CfCYP76AH16 | *C. forskohlii* | AMZ03403.1 |
| CfCYP76AH17 | *C. forskohlii* | AMZ03404.1 |
| CfCYP76AH8 | *C. forskohlii* | AMZ03392.1 |
| CfCYP76AH9 | *C. forskohlii* | AMZ03391.1 |
| ElCYP71D445 | *Euphorbia lathyris* | AMY98418 |
| ElCYP726A27 | *E. lathyris* | AMY98419 |
| EpCYP71D365 | *Euphorbia peplus* | ANO43023 |
| EpCYP726A19 | *E. peplus* | AIM47566.1 |
| EpCYP726A4 | *E. peplus* | AIM47558.1 |
| GbCYP716L | *Ginkgo biloba* | AHF49536.1 |
| IrCYP706V15 | *Isodon rubescens* | WBW48852.1 |
| IrCYP706V16 | *I. rubescens* | WBW48853.1 |
| IrCYP706V2 | *I. rubescens* | UVC58033.1 |
| IrCYP706V3 | *I. rubescens* | UVC58034.1 |
| IrCYP706V4 | *I. rubescens* | UVC58035.1 |
| IrCYP706V6 | *I. rubescens* | UVC58037.1 |
| IrCYP706V7 | *I. rubescens* | UVC58038.1 |
| IrCYP706V8 | *I. rubescens* | UVC58039.1 |
| IrCYP706V9 | *I. rubescens* | UVC58040.1 |
| JcCYP71D495 | *Jatropha curcas* | ANJ20917.1 |
| JcCYP726A20 | *J. curcas* | AIM47550.1 |
| JcCYP726A35 | *J. curcas* | ANJ20916.1 |
| MvCYP71AU87 | *Marrubium vulgare* | QBS13810 |
| NtCYP71D16 | *Nicotiana tabacum* | AAD47832.1 |
| RcCYP726A14 | *Rosmariuns communis* | AIM47545 |
| RcCYP726A15 | *R.communis* | AIM47546 |
| RcCYP726A16 | *R.communis* | AIM47547 |
| RcCYP726A17 | *R.communis* | AIM47548 |
| RcCYP726A18 | *R.communis* | AIM47549 |
| RoCYP76AH22 (RoFS1) | *R. officinalis* | AJQ30187.1 |
| RoCYP76AH23 (RoFS2) | *Rosmariuns officinalis* | AJQ30188.1 |
| RoCYP76AK6 | *R. officinalis* | AOW42544.1 |
| RoCYP76AK7 | *R. officinalis* | AOW42545.1 |
| RoCYP76AK8 | *R. officinalis* | AOW42546.1 |
| SdCYP728D26 | *Salvia divinorum* | QMS79245.1 |
| SdCYP76AH39 | *S. divinorum* | QMS79243.1 |
| SfCYP76AH24 (SfFS) | *Salvia fruticosa* | AJQ30186 |
| SfCYP76AK6 | *S. fruticose;* | AOW42544 |
| SmCYP71D373 | *Salvia miltiorrhiza* | AGN04216.1 |
| SmCYP71D375 | *S. miltiorrhiza* | AWD93836.1 |
| SmCYP76AH1 | *S. miltiorrhiza* | AGN04215 |
| SmCYP76AH3 | *S. miltiorrhiza* | AMB36496 |
| SmCYP76AK1 | *S. miltiorrhiza* | AMB36497 |
| SpCYP71BE52 | *Salvia pomifera* | ALM25794.1 |
| SpCYP76AH24L | *S.pomifera* | ALM25796.1 |
| SpCYP76AK6 | *S.pomifera* | ALM25797.1 |
| SsCYP728DL (SsHTAS) | *Salvia splendens* | XKU00016.1 |
| TcaCYP725A6/T2OH | *Taxus canadensis* | AAS89065.2 |
| TcuCYP725A1/T10OH | *Taxus cuspidata* | AAK00946.1 |
| TcuCYP725A2/T13OH | *T. cuspidata* | AAL23619.1 |
| TcuCYP725A3/T14OH | *T. cuspidata* | AAO66199.1 |
| TcuCYP725A5/T7OH | *T. cuspidata* | AAQ75553.1 |
| VacCYP76BK1 | *Vitex agnus-castus* | AUT77126 |
